# Microstructural Variation upon Introducing Di([2,2′-bithiophen]-5-yl)pyrenes into a Naphthalene Diimide-Based Polymer

**DOI:** 10.1021/acsomega.5c10422

**Published:** 2025-11-06

**Authors:** Kailing Liang, Chih-Hsuan Lin, Yu-Chieh Yeh, Yu-Ying Lai

**Affiliations:** Institute of Polymer Science and Engineering, 33561 National Taiwan University, Taipei 106319, Taiwan

## Abstract

Four pyrene-based conjugated molecules—1,6-bis­(bithiophene)­pyrene (**A**), 1,6-bis­[5-octyl-(2,2′-bithiophen)-5′-yl]­pyrene (**B**), 2,7-bis­[5-octyl-(2,2′-bithiophen)-5′-yl]­pyrene (**C**), and 4,9-bis­[5-octyl-(2,2′-bithiophen)-5′-yl]­pyrene (**D**)—have been designed, synthesized, and blended with poly­[[1,2,3,6,7,8-hexahydro-2,7-bis­(2-octyldodecyl)-1,3,6,8-dioxobenzo­[lmn]­[3,8]­phenanthroline-4,9-diyl]­[2,2′-bithiophene]-5,5′-diyl] (P­(NDI2OD-T2, **P**). The structural difference between **A** and **B** lies in the presence of an alkyl substituent, while **B**, **C**, and **D** are regioisomers. The effects of the alkyl substituent and regioisomerism on the microstructure of **P** have been investigated. Differential scanning calorimetry and ^1^H NMR spectroscopy suggest that alkyl substitution may not play a significant role in determining the crystallization and aggregation of **P**. In contrast, regioisomerism significantly influences these properties. Grazing-incidence X-ray scattering indicates that while the alkyl substituent affects lamellar stacking, regioisomerism plays a crucial role in shaping the polymer’s microstructure. The introduction of pyrene enhances polymer backbone rigidification, likely due to the establishment of naphthalene diimide–pyrene interactions, as supported by density functional theory calculations. Organic field-effect transistor measurements reveal that the blends can exhibit higher electron mobility (μ_e_) than **P**. Linear regression analysis suggests that the crystallization of **P** is correlated with μ_e_. Lastly, the current blending approach is compared with the previous incorporation approach, highlighting the role of molecular degrees of freedom in contributing to the observed differences.

## Graphical abstract

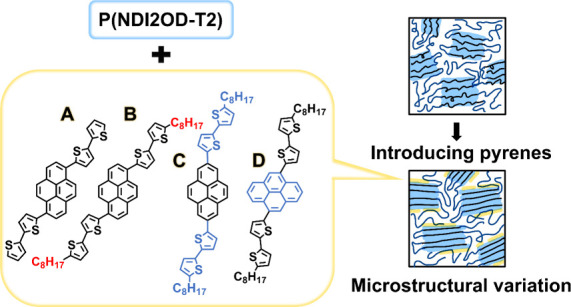

## Introduction

Chain rigidity plays a crucial role in modulating polymer properties. For instance, it influences the glass transition temperature (*T*_g_) and affects polymer stacking, which in turn impacts crystallization. Consequently, adjusting chain rigidity allows for the regulation of a polymer’s mechanical properties.^1–5^ Typically, chain rigidity correlates with incorporating rigid functional groups, where introducing such groups generally increases rigidity.

Polymer microstructure can be divided into crystalline and amorphous phases. The amorphous phase is further subdivided into the mobile amorphous fraction (MAF) and the rigid amorphous fraction (RAF), a concept originally introduced by Wunderlich and colleagues.^6^ In the RAF, although the polymer chains are disordered, they are constrained, resulting in significantly reduced mobility compared to the chains in the MAF. RAF has been observed in numerous polymers, including poly­(oxymethylene),^6^ polyethylene terephthalate,^7–9^ polylactide,^10–15^ polyamide,^16,17^ polyethylene,^18,19^ polypropylene,^20,21^ and polystyrene.^22,23^ with a proposed correlation between chain rigidity and the presence of RAF. It has been suggested that RAF may exist in all semicrystalline polymers.^24–26^ The presence of RAF influences several properties of semicrystalline polymers, such as thermal behavior, mechanical performance, dielectric characteristics, and gas permeability.^7,10,27–29^

Poly­[[1,2,3,6,7,8-hexahydro-2,7-bis­(2-octyldodecyl)-1,3,6,8-dioxobenzo­[lmn]­[3,8] phenanthroline-4,9-diyl]­[2,2′-bithiophene]-5,5′-diyl] (P­(NDI2OD-T2)) (Figure 1), first synthesized by Facchetti and co-workers,^30,31^ has been widely employed in organic field-effect transistors and organic photovoltaics.^31–36^ The naphthalene diimide (NDI) unit in P­(NDI2OD-T2) features a four-fused ring with a quasi-rhombus structure, which is also characteristic of pyrene, a polycyclic aromatic hydrocarbon. However, while both share structural similarities, their electronic properties differ: NDI is an electron-deficient conjugated unit, whereas pyrene is electron-rich. This combination of structural similarity and complementary electronic properties allows for the formation of NDI–pyrene conjugated pairs, which have been documented in the literature.^37–42^ In previous work, our group incorporated pyrene units into P­(NDI2OD-T2) to synthesize a series of random copolymers (Figure 1a).^43^ The incorporation of pyrene not only enhances the rigidity of the polymer chains but also leads to notable changes in the polymer’s microstructure. At higher pyrene content, NDI–pyrene interactions dominate, giving rise to a unique polymorph. With moderate pyrene content, the crystallites fragment, leading to an increase in the RAF. Our earlier study highlights an effective strategy to regulate the microstructure of P­(NDI2OD-T2).

**Figure 1:**
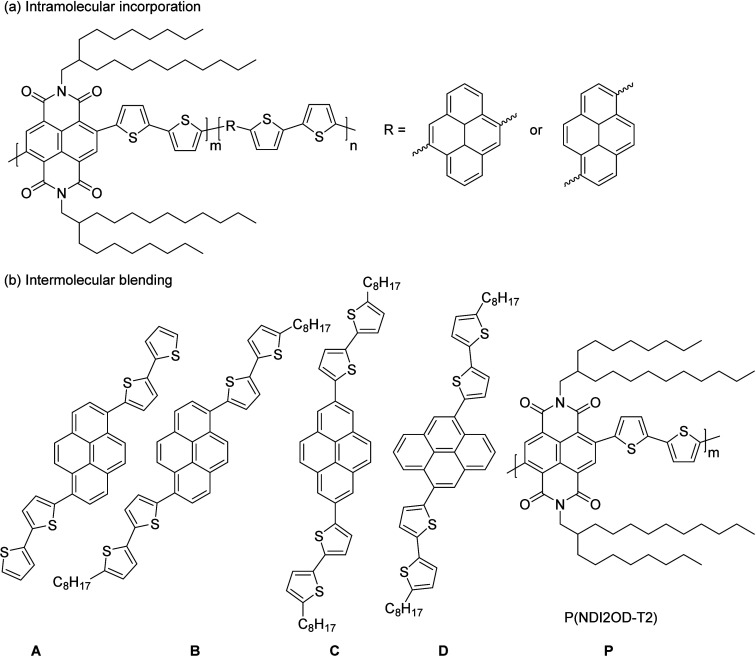
Illustrations showing (a) pyrene units incorporated into P­(NDI2OD-T2) and (b) pyrene additives blended with P­(NDI2OD-T2).

While NDI–pyrene interactions have been recognized in molecular systems, their potential in polymeric systems remains largely unexplored. In this study, four pyrene-based conjugated molecules—1,6-bis­(bithiophene)­pyrene (**A**), 1,6-bis­[5-octyl-(2,2′-bithiophen)-5′-yl]­pyrene (**B**), 2,7-bis­[5-octyl-(2,2′-bithiophen)-5′-yl]­pyrene (**C**), and 4,9-bis­[5-octyl-(2,2′-bithiophen)-5′-yl]­pyrene (**D**)—have been designed, synthesized, and blended with P­(NDI2OD-T2) (Figure 1b). The structural difference between **A** and **B** lies in the presence of an octyl substituent, while **B**, **C**, and **D** are regioisomers with different linkage modes between the pyrene and bithiophene units. The effects of the alkyl substituent and regioisomerism on the microstructure of P­(NDI2OD-T2) have been investigated. Furthermore, in the previous study, pyrene units were incorporated into P­(NDI2OD-T2), which can be considered intramolecular incorporation. In contrast, in this study, pyrene additives are blended with P­(NDI2OD-T2), which can be considered intermolecular blending. A comparison has been made between intramolecular incorporation and intermolecular blending. Overall, this study elucidates the microstructural variations that occur upon introducing di­([2,2′-bithiophen]-5-yl)­pyrene derivatives into P­(NDI2OD-T2).

## Results and Discussion

### Synthesis

P­(NDI2OD-T2), hereafter referred to as **P**, was synthesized following our previously established protocol.^32,43^ High-temperature gel permeation chromatography determined the number-average molar mass (*M*_n_) of **P** to be 18.3 kg mol^–1^, with a weight-average molar mass (*M*_w_) of 40.3 kg mol^–1^, resulting in a dispersity (*Đ*) of 2.19. The synthesis of four di­([2,2′-bithiophen]-5-yl)­pyrenes is depicted in Scheme 1. The discussion begins with the synthesis of 5-trimethylstannyl-2,2′-bithiophene (**2**) and 5-octyl-5′-(tri-*n*-butylstannyl)-2,2′-bithiophene (**5**). Commercially available 2,2′-bithiophene (**1**) was deprotonated by *n*-butyllithium in THF and then reacted with trimethyltin chloride to give 5-trimethylstannyl-2,2′-bithiophene (**2**). On the other hand, the Friedel–Crafts acylation of **1** with octanoyl chloride resulted in the formation of 1-[(2,2′-bithiophene)-5-yl]­octan-1-one (**3**). The carbonyl group of **3** was then reduced to a methylene group via a Wolff–Kishner reduction, producing 5-octyl-2,2′-bithiophene (**4**). Subsequently, **4** was deprotonated by *n*-butyllithium and reacted with tributyltin chloride to furnish 5-octyl-5′-(tri-*n*-butylstannyl)-2,2′-bithiophene (**5**).

**Scheme 1:**
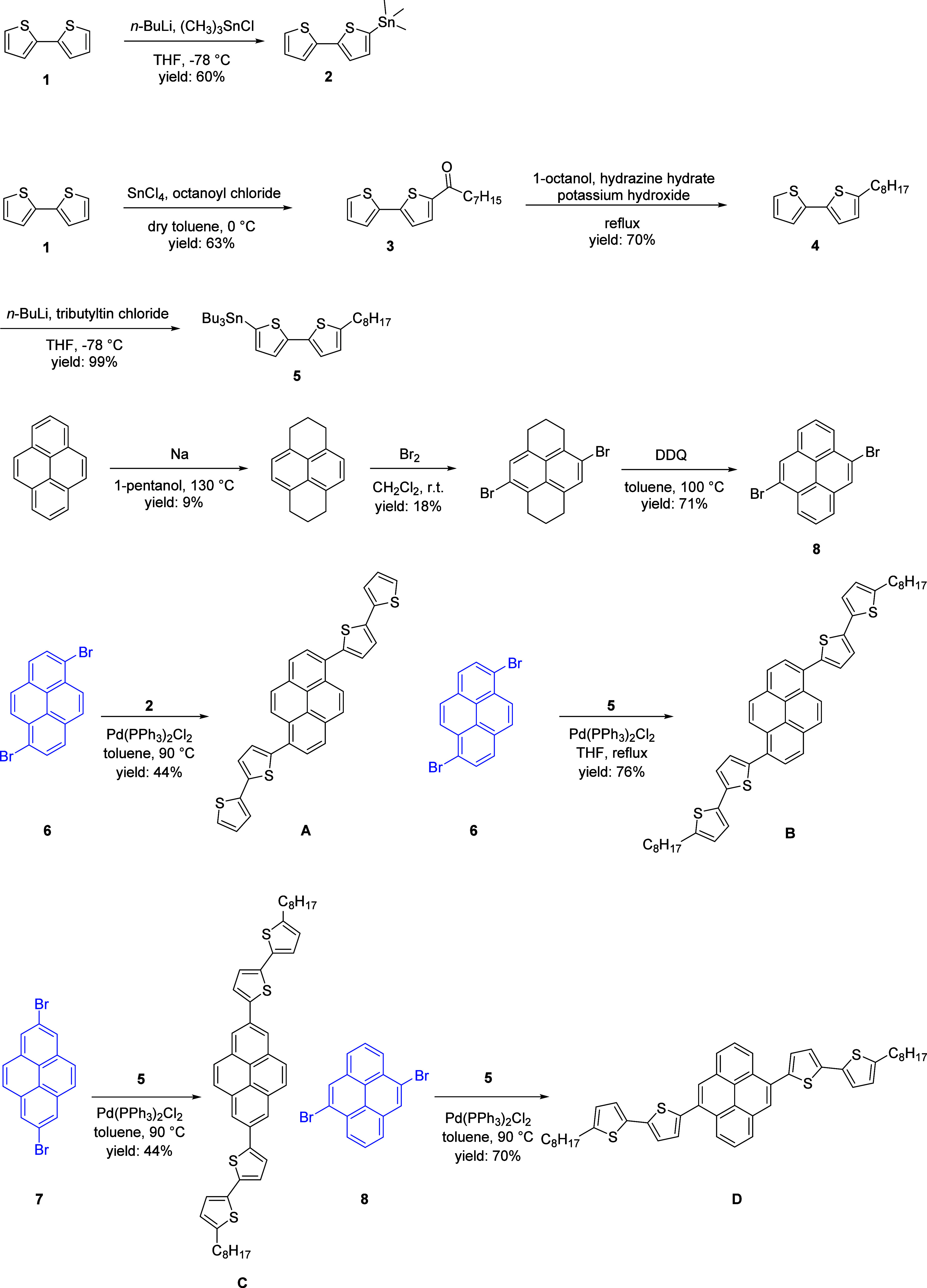
Synthetic Routes to **A**, **B**, **C**, and **D**

1,6-Dibromopyrene (**6**) and 2,7-dibromopyrene (**7**) were acquired from commercial sources. Birch reduction followed by bromination and dehydrogenation led to the formation of 4,9-dibromopyrene (**8**).^43^ Stille coupling of **6** with **2** or **5** was carried out to afford compound **A** or **B** in 44% or 76% yields, respectively. Similarly, Stille coupling of **7** or **8** with **5** was performed to give compound **C** or **D** in 44% or 70% yields, respectively. The synthesized compounds were characterized by nuclear magnetic resonance (NMR) and mass spectrometer. Detailed spectral data and analysis are shown in the Supporting Information.

### Thermogravimetric Analysis

Thermogravimetric analysis (TGA) evaluated the thermal stability of **P**, **A**, **B**, **C**, and **D**. The thermal decomposition temperature (*T*_d_), defined by a 5% weight loss, was measured at 455 °C for **P**, 388 °C for **A**, 436 °C for **B**, 410 °C for **C**, and 396 °C for **D** (Figure S1, Supporting Information). These values suggest that all the molecules exhibit good thermal stability.

### UV–Vis Spectroscopy

**P** was blended with **A**, **B**, **C**, and **D**, respectively, using three different weight percentages: 3%, 5%, and 10% of each pyrene additive. The effects of the additives were examined from two perspectives: alkyl substitution and regioisomerism. The impact of alkyl substitution was assessed by comparing the **A** and **B** blends, while the effect of regioisomerism was investigated by comparing the **B**, **C**, and **D** blends.

All UV–vis absorption spectra in thin films are presented in Figure S2, Supporting Information. **P** exhibited two absorption bands, with maximal absorbance (λ_max_) at 380 and 700 nm. According to previous studies, the band at 700 nm is attributed to intramolecular charge transfer, while the band at 380 nm corresponds to π–π* transitions.^43,44^ After introducing pyrene additives to **P**, a bathochromic shift in the λ_max_ at 700 nm was observed (Figure 2a). Specifically, λ_max_ was 712 nm for **P**+10%**A**, 706 nm for **P**+10%**B**, 704 nm for **P**+10%**C**, and 716 nm for **P**+10%**D**. Figure 2b illustrates the absorption spectra of blends with 3% pyrene additives. A shoulder at 658 nm, absent in pure **P**, was detected in these blending systems. Notably, the absorbance of additives **A**, **B**, **C**, or **D** is below 600 nm, indicating that the bathochromic shift in λ_max_ and the appearance of the shoulder result from interactions between **P** and the additives rather than direct absorption by the additives. This bathochromic shift may also be associated with J-type aggregation of the polymers.^45,46^

**Figure 2:**
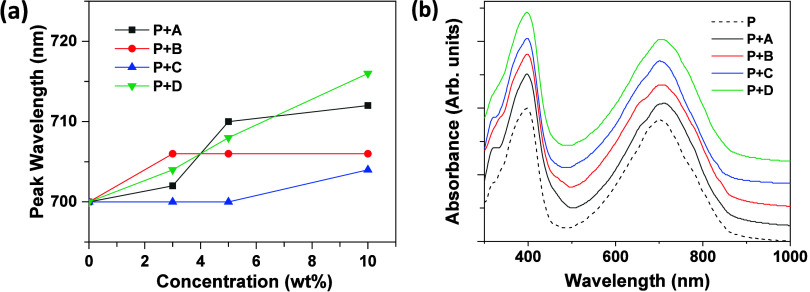
(a) Variation in the λ_max_ at 700 nm after introducing various amounts of pyrene additives to **P**. (b) Absorption spectra of blends with 3% additives.

A comparison of **P**+10%**A** and **P**+10%**B** revealed that **A**, which lacks alkyl chains, exhibited a longer λ_max_ (712 nm) compared to **B**, which has octyl chains (λ_max_ = 706 nm). Additionally, among **P**+10%**B**, **P**+10%**C**, and **P**+10%**D**, **D**, with the 4,9 linking mode, resulted in the longest λ_max_ (716 nm).

### Differential Scanning Calorimetry

To evaluate the phase transition of the blends, differential scanning calorimetry (DSC) was employed. The DSC results are summarized in Table 1, and the calorimetric curves are included in Figure S3, Supporting Information. Pristine **P** exhibited a melting temperature (*T*_m_) of ∼312 °C, while **A** exhibited a *T*_m_ of ∼299 °C, **B** exhibited two *T*_m_s at ∼248 and 226 °C, **C** exhibited three *T*_m_s at ∼366, 218, and 142 °C, and **D** exhibited two *T*_m_s at ∼152 and 125 °C. For crystallization temperatures (*T*_c_), pristine **P** exhibited a *T*_c_ of ∼254 °C, **A** exhibited a *T*_c_ of ∼273 °C, **B** exhibited two *T*_c_s at ∼245 and 210 °C, **C** exhibited two *T*_c_s at ∼363 and 211 °C, and **D** exhibited two *T*_c_s at ∼147 and 81 °C. These results reveal that di­([2,2′-bithiophen]-5-yl)­pyrene derivatives, with variations in side chains and regioisomerism, exhibit distinct phase transition behaviors.

**Table 1:** DSC Results for the Blends

	*T*_m_ (°C)	Δ*H*_m_ (J/g)	*T*_c_ (°C)	Δ*H*_c_ (J/g)
**P**	312	21	254	13
**A**	299	94	273	84
**P**+3%**A**	323	19	292	16
**P**+5%**A**	314	20	279	13
**P+**10%**A**	309/283	7/5	280	11
**B**	248/226	9/52	245/210	8/47
**P**+3%**B**	324	14	294	14
**P**+5%**B**	316	17	261	13
**P**+10%**B**	311/283	8/5	281	11
**C**	366/218/142	16/54/16	363/211	14/51
**P**+3%**C**	325	28	288	16
**P**+5%**C**	324/216	14/0.4	285	13
**P**+10%**C**	319/216	11/3	279	14
**D**	152/125	2/39	147/81	3/36
**P**+3%**D**	317	20	287	16
**P**+5%**D**	315	21	287	15
**P**+10%**D**	311	16	281	13

In the **A** blends, **P**+3%**A** and **P**+5%**A** exhibited *T*_m_s of ∼323 °C and ∼314 °C, respectively, which were higher than those of pristine **P** or **A**. These results indicate that the intermolecular interactions within the crystallites might be stronger in **P**+3%**A** and **P**+5%**A** compared to pristine **P** and **A**, which may be associated with the formation of NDI–pyrene conjugated pairs. **P**+10%**A** exhibited two *T*_m_s, at ∼309 °C and ∼283 °C, suggesting the occurrence of phase separation. The crystallites with a higher *T*_m_ of ∼309 °C may be more polymer-rich than those with a lower *T*_m_ of ∼283 °C. Furthermore, the *T*_c_s in the **A** blends, were all higher than the *T*_c_ of **P**, suggesting that the blends crystallized faster than **P**. This may be due to **A** acting as a nucleating agent to promote the crystallization of **P**. Additionally, the variation in the enthalpy of melting (Δ*H*_m_) revealed that the **A** blends all exhibited lower Δ*H*_m_ values than **P**, suggesting that the introduction of **A** into **P** may reduce the overall crystallinity of **P**.

Similar results were observed for the **B** blends. **P**+3%**B** and **P**+5%**B** exhibited higher *T*_m_s of ∼324 °C and ∼316 °C, respectively, compared to pristine **P** or **B**, indicating stronger intermolecular interactions within the crystallites of **P**+3%**B** and **P**+5%**B** than in pristine **P** and **B**. **P**+10%**B** exhibited two *T*_m_s, at ∼311 °C and ∼283 °C, suggesting the occurrence of phase separation. The *T*_c_s of the **B** blends were all higher than the *T*_c_ of **P**, suggesting that **B** may act as a nucleating agent, promoting the crystallization of **P**. Additionally, the variation in Δ*H*_m_ revealed that all the **B** blends exhibited lower Δ*H*_m_ values than **P**, suggesting that the introduction of **B** into **P** may reduce the overall crystallinity of **P**.

In the **C** blends, two *T*_m_s were observed for both **P**+5%**C** (∼324 °C and ∼216 °C) and **P**+10%**C** (∼319 °C and ∼216 °C), suggesting phase separation in both **P**+5%**C** and **P**+10%**C**. In contrast, for the **A** and **B** blends, phase separation occurred only at a 10 wt % additive level. **P**+3%**C** exhibited a single *T*_m_ of ∼325 °C with a Δ*H*_m_ of ∼28 J g^–1^, both of which were higher than the values for pristine **P**. The *T*_c_s of all **C** blends were higher than the *T*_c_ of **P**, suggesting that **C** may act as a nucleating agent to promote the crystallization of **P**. Additionally, the variation in Δ*H*_m_ revealed that all the **C** blends, except for **P**+3%**C**, exhibited lower Δ*H*_m_ values than **P**, suggesting that the introduction of **C** into **P** may reduce the overall crystallinity of **P**.

No phase separation was observed in the **D** blends, which may indicate that **D** has lower crystallinity compared to **A**, **B**, and **C**. **P** + 3%**D** and **P**+5%**D** exhibited higher *T*_m_s of ∼317 °C and ∼315 °C, respectively, compared to pristine **P** or **D**, indicating that the intermolecular interactions within the crystallites of **P**+3%**D** and **P**+5%**D** may be stronger than those in pristine **P** and **D**. In contrast, **P**+10%**D** exhibited a slightly lower *T*_m_ of ∼311 °C than **P**. Similar to the **A**, **B**, and **C** blends, the *T*_c_s of all **D** blends were higher than the *T*_c_ of **P**, suggesting that **D** may act as a nucleating agent to promote the crystallization of **P**. The Δ*H*_m_ values were ∼20 J g^–1^ for **P**+3%**D**, ∼21 J g^–1^ for **P**+5%**D**, and ∼16 J g^–1^ for **P**+10%**D**. The variation in Δ*H*_m_ was less significant in the **D** blends than in the **A**, **B**, and **C** blends.

The variation trend between the **A** and **B** blends was rather similar, suggesting that alkyl substitution might not play a significant role in determining the crystallization of **P**. However, comparison of the **B**, **C**, and **D** blends revealed distinct variation trends, especially regarding phase separation, which occurred at 10 wt % for **B**, at both 5 and 10 wt % for **C**, and was not detected for **D**. These results suggest that regioisomerism plays a significant role in determining the crystallization of **P**.

### Rigid Amorphous Fraction

According to previous work, a shoulder region preceding the *T*_m_ in DSC thermograms for NDI-based polymers may result from the RAF.^43^ Shoulder regions were more visible in the blends of **P**+3%**C**, **P**+5%**C**, **P**+3%**D**, and **P**+5%**D**. Using the previous curve-fitting method, their endothermic DSC curves near the *T*_m_ were fitted with a combination of two functions: the first function describes the heat flow of **P**, while the second is a sigmoid curve representing the glass transition of the RAF, yielding the change in heat capacity of the polymer (*W*_s_). After fitting, the *W*_s_ values were 0.48 W g^–1^ for **P**+3%**C**, 0.17 W g^–1^ for **P**+5%**C**, 0.36 W g^–1^ for **P**+3%**D**, and 0.40 W g^–1^ for **P**+5%**D**. These results suggest that **P**+3%**C** might exhibit the most pronounced RAF among the blends. No apparent shoulder corresponding to RAF was detected in the **A** and **B** blends, suggesting that regioisomerism plays a significant role in RAF formation. In contrast, **C** and **D** appear to promote RAF formation more effectively than **A** and **B.**

### ^1^H NMR Spectroscopy

Neher and co-workers used ^1^H NMR spectroscopy to study the aggregation of **P**, finding that the chemical shifts of NDI protons in **P** split into two regions: a downfield shift corresponding to unaggregated polymer chains and an upfield shift corresponding to stacked or aggregated polymer chains. In this study, **P** and all blends were examined using ^1^H NMR spectroscopy, with the corresponding spectra shown in Figure S4, Supporting Information. Based on this characterization, the chemical shift at ∼8.7–8.9 ppm was attributed to unaggregated polymer chains, while the shift at ∼8.4–8.6 ppm was attributed to stacked chains. The proportion of stacked chains was estimated from the integration values in the ^1^H NMR spectra, and the variation in this proportion is illustrated in Figure 3.

**Figure 3:**
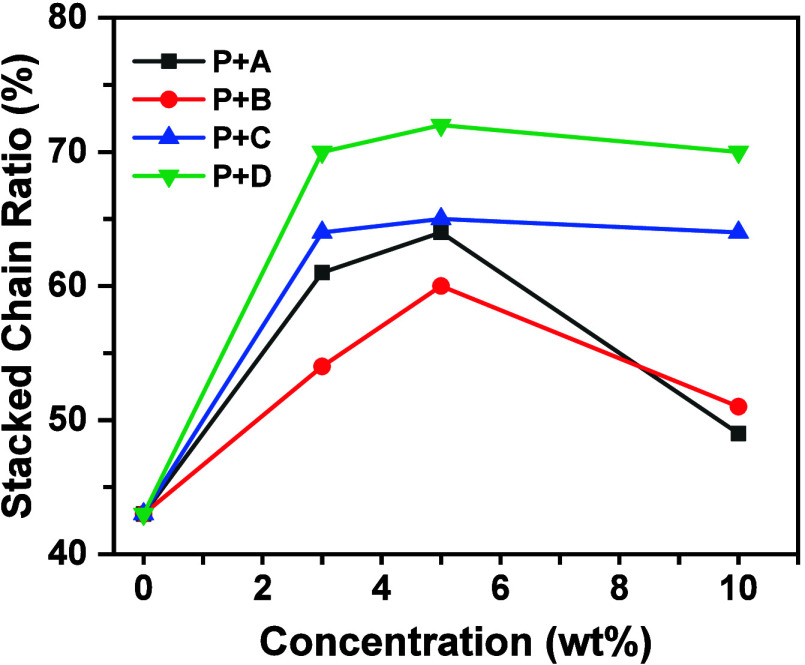
Stacked-chain ratio of **P** blended with varying amounts of pyrene additives.

For the **A** blends, the stacked-chain proportion increased from 43% (0 wt %) to 61% (3 wt %), 64% (5 wt %), and then decreased to 49% (10 wt %). For the **B** blends, the proportion increased from 43% (0 wt %) to 54% (3 wt %), 60% (5 wt %), and 51% (10 wt %). For the **C** blends, the proportion rose from 43% (0 wt %) to 64% (3 wt %), 65% (5 wt %), and remained at 64% (10 wt %). For the **D** blends, it increased from 43% (0 wt %) to 70% (3 wt %), 72% (5 wt %), and remained at 70% (10 wt %).

Although the values differed, the variation trend between the **A** and **B** blends was quite similar, suggesting that alkyl substitution may not play a significant role in determining **P**’s aggregation. The drop in the stacked-chain proportion at 10 wt % may be associated with the phase separation observed in DSC, although this drop was less pronounced for the **C** and **D** blends. Both **C** and **D** blends exhibited a higher proportion of stacked chains than the **A** and **B** blends, with the **D** blends showing the highest proportion among all. A comparison of the **B**, **C**, and **D** blends suggests that regioisomerism may play a significant role in determining the aggregation behavior of **P**.

### Grazing-Incidence X-ray Scattering

To further investigate the microstructure of the blends, grazing-incidence X-ray scattering (GIXS) was performed. The GIXS image of **P** revealed a signal along the vertical axis at *q*_*z*_ ≈ 1.6 Å^–1^, corresponding to the out-of-plane π-stacking, identified as the (010) reflection (Figure S5, Supporting Information).^43,47^ Along the in-plane direction, two signals were observed at *q*_r_ ≈ 0.25 Å^–1^ and *q*_r_ ≈ 0.45 Å^–1^, corresponding to the lamellar stacking and the polymer backbone, designated as the (100) and (001) reflections, respectively.^48^ Scherrer’s equation was applied to one-dimensional diffractograms to estimate the coherence lengths of stacking orders.^49,50^ The coherence length of the π-stacking (*L*_c, π_) was ∼21 Å, that of the lamellar stacking (*L*_c,L_) was ∼166 Å, and that of the polymer backbone (*L*_c,B_) was ∼95 Å (Table S1, Supporting Information). Overall, the data revealed that **P** exhibited a face-on configuration in films, consistent with previous studies.

All blends exhibited a face-on configuration in thin films, similar to pristine **P**, and no other polymorphs were detected. However, the coherence lengths, particularly *L*_c,L_ and *L*_c,B_, were significantly influenced by the introduction of pyrene additives. Variations in *L*_c, π_, *L*_c,L_, and *L*_c,B_ across the blends are shown in Figure 4. The *L*_c,π_ values were ∼21 Å for **P**, ∼16 Å for **P**+3%**A**, ∼17 Å for **P**+5%**A**, and ∼17 Å for **P**+10%**A**, indicating that the introduction of **A** decreased *L*_c, π_ by approximately one π-stacking spacing. The *L*_c,L_ values were ∼166 Å for **P**, ∼119 Å for **P**+3%**A**, ∼128 Å for **P**+5%**A**, and ∼119 Å for **P**+10%**A**, indicating that the introduction of **A** significantly reduced *L*_c,L_. Lamellar stacking, which refers to the separation of conjugated and aliphatic moieties, was shortened, likely due to compound **A** lacking aliphatic side chains. For *L*_c,B_, the values were ∼95 Å for **P**, ∼126 Å for **P**+3%**A**, ∼109 Å for **P**+5%**A**, and ∼89 Å for **P**+10%**A**. The elongated *L*_c,B_ values suggested that the polymer backbone was rigidified, likely due to the development of NDI–pyrene interactions. However, the elongation of *L*_c,B_ was less pronounced for **P**+10%**A**, which might be attributed to phase separation, as suggested by DSC analysis.

**Figure 4:**
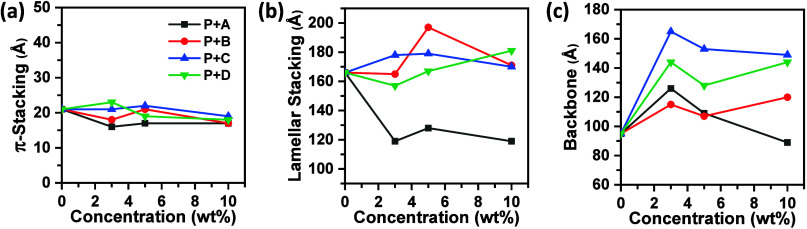
Variation in coherence length of (a) π-stacking (*L*_c,π_), (b) lamellar stacking (*L*_c,L_), and (c) backbone (*L*_c,B_) for P blended with 1, 3, 5, and 10 wt % of pyrene additives.

For the **B** blends, the *L*_c,π_ values were ∼21 Å for **P**, ∼18 Å for **P**+3%**B**, ∼21 Å for **P**+5%**B**, and ∼17 Å for **P**+10%**B**. Although some variation was observed, the introduction of **B** appeared to have an insignificant effect on *L*_c,π_. Similar conclusions were drawn for the **C** and **D** blends.

The *L*_c,L_ values were ∼166 Å for **P**, ∼165 Å for P+3%**B**, ∼197 Å for P+5%**B**, and ∼171 Å for P+10%**B**, indicating that *L*_c,L_ either remained comparable to or increased relative to pristine **P**. Similar conclusions were drawn for the **C** and **D** blends. A comparison of the *L*_c,L_ values for the **A** and **B** blends highlights the significant role of the alkyl substituent in determining *L*_c,L_.

For *L*_c,B_, the values were ∼95 Å for **P**, ∼115 Å for **P**+3%**B**, ∼107 Å for **P**+5%**B**, and ∼120 Å for **P**+10%**B**. The elongated *L*_c,B_ values suggested that the polymer backbone was rigidified, likely as a result of the establishment of NDI–pyrene interactions. Similar conclusions were drawn for the **C** and **D** blends.

Overall, GIXS suggests that the alkyl substituent has a significant impact on lamellar stacking. A comparison of the **B**, **C**, and **D** blends reveals that regioisomerism plays a significant role in determining the microstructure, particularly influencing the polymer backbone.

### DFT Calculations

To investigate the potential interaction mechanisms between **P** and pyrene additives **A**, **B**, **C**, and **D**, density functional theory (DFT) calculations were performed. To reduce computational cost, **P** was simplified as a dimer, with the 2-octyldodecyl group replaced by a 2-methylpropyl group. Similarly, the octyl groups in **B**, **C**, and **D** were replaced by ethyl groups. The optimized geometries of the simplified models at the ωB97XD/6–311G­(d,p) level of theory are illustrated in Figure 5.

**Figure 5:**
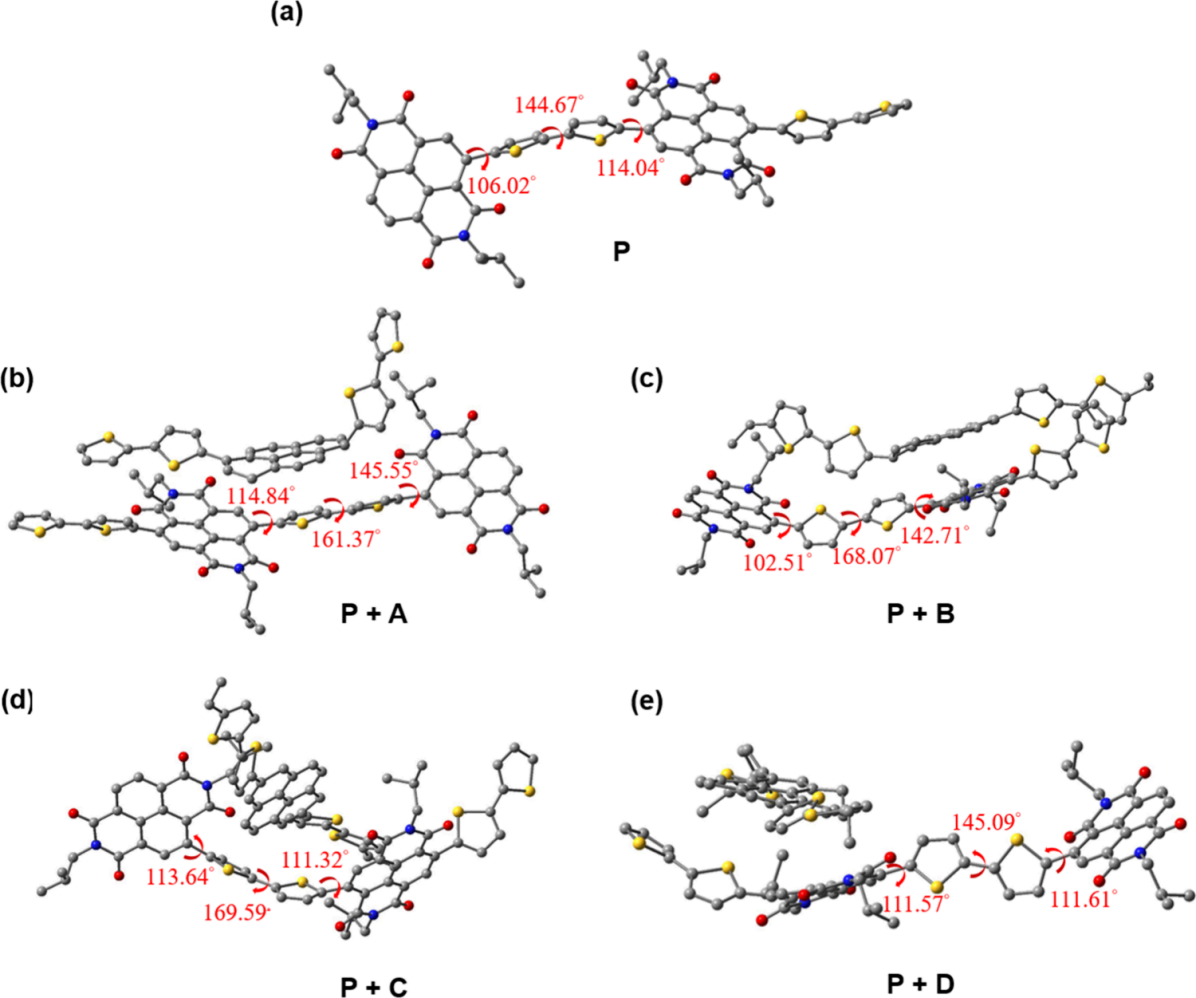
Optimized geometries of (a) the **P** model compound and its interaction with (b) **A**, (c) **B**, (d) **C**, and (e) **D**.

The dihedral angles between the NDI and thiophene unit, between the two thiophene units, and between the thiophene and NDI units in the **P** model compound were calculated to be 106°, 145°, and 114°, respectively. Upon interaction with **A**, these dihedral angles increased, suggesting that the conjugated backbone of **P** becomes more planar. Similar results were observed for **P** interacting with **B** and **C**, indicating that the NDI–pyrene interaction promotes increased planarity of the conjugated units in **P**. This enhanced planarity is expected to contribute to the rigidity of the polymer backbone.

The computational results align with the GIXS findings, indicating that the introduction of pyrene compounds leads to an elongated *L*_c,B_, which is associated with the rigidification of the polymer backbone. The increased planarity of the conjugated backbone, as revealed by DFT calculations, supports the observed structural changes in the blends and highlights the role of NDI–pyrene interactions in enhancing polymer rigidity.

### Organic Field-Effect Transistors

Organic field-effect transistors (OFETs) with a bottom-gate/top-contact configuration were fabricated using **P** blended with pyrene additives **A**, **B**, **C**, and **D** as the semiconductive layer to evaluate their electrical characteristics. The electron mobility (μ_e_), threshold voltage (*V*_th_), and on/off current ratio were evaluated, with detailed results summarized in Table S2 and Figure S6, Supporting Information. The average and maximum μ_e_ values for each blend are demonstrated in Figure 6.

**Figure 6:**
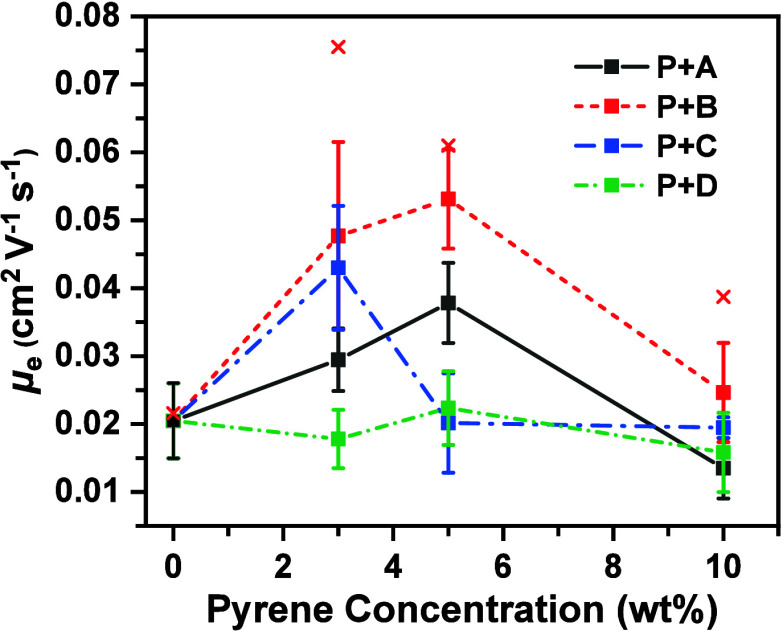
Electron mobility of **P** blended with pyrene additives **A**, **B**, **C**, and **D** at various concentrations. Box symbols represent the average mobility, cross symbols indicate the maximum mobility, and error bars denote the standard deviation.

The pristine **P** exhibited an average μ_e_ of 0.020 cm^2^ V^–1^ s^–1^ and a maximal μ_e_ of 0.027 cm^2^ V^–1^ s^–1^. The average μ_e_ was 0.030 cm^2^ V^–1^ s^–1^ for **P**+3%**A**, 0.038 cm^2^ V^–1^ s^–1^ for **P**+5%**A**, and 0.013 cm^2^ V^–1^ s^–1^ for **P**+10%**A**. The maxima μ_e_ was 0.049 cm^2^ V^–1^ s^–1^ achieved by **P**+5%**A**. The introduction of **A** into **P** can increase the μ_e_. Moreover, a decline in the μ_e_ was observed for **P**+10%**A**, which might be associated with the occurrence of phase separation, as revealed by DSC.

For the **B** blends, the average μ_e_ was 0.048 cm^2^ V^–1^ s^–1^ for **P**+3%**B**, 0.053 cm^2^ V^–1^ s^–1^ for **P**+5%**B**, and 0.025 cm^2^ V^–1^ s^–1^ for **P**+10%**B**. The maxima μ_e_ was 0.076 cm^2^ V^–1^ s^–1^ achieved by **P**+3%**B**. The introduction of **B** into **P** can increase the μ_e_. Moreover, a drop in the μ_e_ was observed for **P**+10%**B**, which might be associated with the occurrence of phase separation, as revealed by DSC.

For the **C** blends, the average μ_e_ was 0.043 cm^2^ V^–1^ s^–1^ for **P**+3%**C**, 0.020 cm^2^ V^–1^ s^–1^ for **P**+5%**C**, and 0.019 cm^2^ V^–1^ s^–1^ for **P**+10%**C**. The maxima μ_e_ was 0.054 cm^2^ V^–1^ s^–1^ achieved by **P**+3%**C**. The introduction of **C** into **P** can increase the μ_e_. Moreover, a drop in the μ_e_ was observed for **P**+5%**C** and **P**+10%**C**, both of which exhibited phase separation, as revealed by DSC.

For the **D** blends, the average μ_e_ was 0.018 cm^2^ V^–1^ s^–1^ for **P**+3%**D**, 0.022 cm^2^ V^–1^ s^–1^ for **P**+5%**D**, and 0.017 cm^2^ V^–1^ s^–1^ for **P**+10%**D**. The values were comparable to that (0.020 cm^2^ V^–1^ s^–1^) of the pristine **P**. It seemed that the introduction of **D** into **P** did not increase the μ_e_.

DSC revealed that the variation in Δ*H*_m_ was less significant in the **D** blends than in the **A**, **B**, and **C** blends, indicating that the total interaction of **P** with **D** might be less pronounced than that with **A**, **B**, or **C**. This observation aligns with the experimental results, which showed that the μ_e_ was relatively insensitive to the introduction of **D.**

Linear regression analysis was conducted between the average μ_e_ and the Δ*H*_m_ determined from DSC for all blends. The resulting R^2^ values were ∼0.96 for the **A** blends, ∼0.84 for the **B** blends, ∼0.99 for the **C** blends, and ∼0.76 for the **D** blends (Figure 7). A similar analysis was performed between the average μ_e_ and the stacked-chain proportion determined from ^1^H NMR spectroscopy, yielding R^2^ values of ∼0.98 for the **A** blends, ∼0.73 for the **B** blends, ∼0.22 for the **C** blends, and ∼0.96 for the **D** blends (Figure S7, Supporting Information). These regression analyses suggest that Δ*H*_m_ may be correlated with the average μ_e_. The results indicate that the crystallization of **P** likely plays a significant role in determining μ_e_. It should be noted that apparent RAF was detected only in the blends of **P**+3%**C**, **P**+5%**C**, **P**+3%**D**, and **P**+5%**D**. Consequently, establishing a direct correlation between RAF and the OFET characteristics is not feasible. This observation also suggests that RAF may not play a critical role in differentiating the OFET performance among the blends.

**Figure 7:**
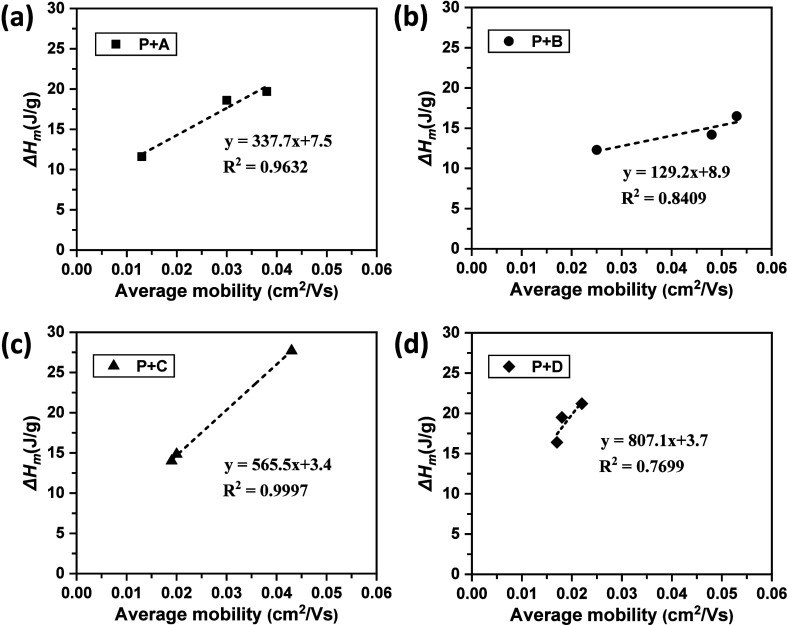
Linear regression analyses of the average μ_e_ versus the Δ*H*_m_ for (a) **A** blends, (b) **B** blends, (c) **C** blends, and (d) **D** blends.

### Comparison with the Incorporation Approach

As mentioned in Introduction, in the previous study, two types of pyrenes are incorporated randomly into **P**, which can be considered intramolecular incorporation (Figure 1a). The growth in the pyrene quantity in a polymer results in the reduction of π-order. For 5 and 10 mol % pyrene incorporation, form I crystallites, that results from **P**, are fragmented, increasing the RAF regions. For 20 mol % pyrene content, a form II polymorph that is different from the stacking structure of **P** is established. Further details can be found in reference 43.

In the current study, pyrene additives are blended with **P**, which can be considered intermolecular blending. The reduction of π-order is not significant for the blending approach. The RAF is only visible in the **C** and **D** blends. Although the crystallization and aggregation are affected significantly by the introduction of pyrene, no new stacking is identified. The results between the incorporation and blending approaches are quite different. In the incorporation approach, pyrenes are incorporated into **P**, and thus the degrees of freedom of pyrenes are restricted. In contrast, in the blending approach, pyrenes are blended with **P** and thus they should possess higher freedom than those in the incorporation approach. We reckon that degrees of freedom of pyrenes might result in the observed differences.

## Conclusions

Four pyrene-based conjugated molecules—**A**, **B**, **C**, and **D**—has been designed and synthesized. The structural difference between **A** and **B** lies in the presence of an alkyl substituent, while **B**, **C**, and **D** are regioisomers. The effects of the alkyl substituent and regioisomerism on the microstructure of **P** has been investigated. TGA reveals that **A**, **B**, **C**, **D**, and **P** exhibit *T*_d_ above 390 °C, indicating good thermal stability. **P** has been blended with **A**, **B**, **C**, and **D** at three different weight percentages: 3%, 5%, and 10%.

UV–vis absorption spectroscopy suggests that the bathochromic shift in λ_max_ and the appearance of a shoulder in **P** after blending may result from interactions between **P** and the pyrene additives. DSC and ^1^H NMR spectroscopy indicate that alkyl substitution does not significantly influence the crystallization and aggregation of **P**, whereas regioisomerism plays a crucial role. GIXS analysis shows that while the alkyl substituent affects lamellar stacking, regioisomerism strongly impacts the polymer microstructure. The introduction of pyrene enhances polymer backbone rigidification, likely due to the establishment of naphthalene diimide–pyrene interactions.

DFT calculations suggest increased planarity of the conjugated backbone of **P** after blending, aligning with the polymer backbone rigidification observed in GIXS. OFET measurements reveal that the blends can exhibit higher μ_e_ than pristine **P**. Linear regression analyses suggest a correlation between the Δ*H*_m_ estimated from DSC and the average μ_e_. These findings indicate that the crystallization of **P** could play a significant role in determining μ_e_.

Lastly, a comparison between the current blending approach and the previous incorporation approach highlights the role of molecular degrees of freedom in the observed differences. Overall, this study elucidates the microstructural variations that occur upon introducing di­([2,2′-bithiophen]-5-yl)­pyrene derivatives into **P** and paves the way for utilizing conjugated pairs to regulate the microstructure of conjugated polymers.

## Supplementary Material



## Notes

Results for general descriptions, synthesis, TGA, UV–vis spectroscopy, DSC, ^1^H NMR spectroscopy, GIXS, DFT calculations, OFET, linear regression analysis, and NMR and mass spectra (PDF)
