# Supplementary material for: Microstructural Variation upon Introducing Di([2,2′-bithiophen]-5-yl)pyrenes into a Naphthalene Diimide-Based Polymer
Source: ACS Omega. 2025 Nov 6;10(45):55105–14. doi: 10.1021/acsomega.5c10422 (PMC12631666; doi:10.1021/acsomega.5c10422)
Supplement: Supplementary file 1 [file ao5c10422_si_001.pdf]

Supporting Information

Microstructural Variation upon Introducing Di([2,2'-bithiophen]-5-yl)pyrenes into a Naphthalene Diimide-Based Polymer

*Kailing Liang,[‡] Chih-Hsuan Lin,[‡] Yu-Chieh Yeh, and Yu-Ying Lai**

Institute of Polymer Science and Engineering, National Taiwan University, Taipei 106319, Taiwan

Email: yuyinglai@ntu.edu.tw

Contents

1. General descriptions 2
2. Synthesis 3
3. TGA 10
4. UV-vis spectroscopy 11
5. DSC 12
6. ^{1}H NMR spectroscopy 14
7. GIXS 15
8. DFT calculations 18
9. OFET 19
10. Linear regression analysis 23
11. NMR and mass spectra 24

1. General descriptions

NMR. ^{1}H NMR spectra were recorded using a Bruker AVIII HD-600 MHz spectrometer at the Instrumentation Center of National Taiwan Normal University.

HT-GPC. High-temperature gel permeation chromatography (HT-GPC) measurements were performed using an HLC-8321GPC/HT high-temperature system equipped with an RI detector at the Department of Applied Chemistry, National Yang Ming Chiao Tung University. The system was integrated with Tosoh Bioscience LLC TSKgel GMHIHR-HIHT2 mixed-bed columns, and 1,2,4-trichlorobenzene was used as the eluent at 160 °C.

MS. Mass spectrometer (MS) experiments were conducted using a Bruker New ultrafleXtreme™ mass spectrometer at the Mass Spectrometry Facility, Institute of Chemistry, Academia Sinica.

EA. Elemental analysis (EA) was performed using an Elementar Vario EL cube (for NCSH) at the Precious Instrument Center of National Taiwan University.

Thermal properties. Thermogravimetric analysis (TGA) was conducted using a TA Instruments Q-50 at a heating rate of 10 °C min^{-1} up to 800 °C. Decomposition temperature (T_d) is defined by the temperature of 5% weight loss of the initial mass. Differential scanning calorimetry measurements (DSC) were performed using a TA Instruments Q-20 system under a nitrogen atmosphere at a heating/cooling rate of 10 °C min^{-1}.

Optical spectroscopy. UV-vis absorption spectra were recorded using a UV Hitachi U4100 spectrophotometer over the wavelength range of 300–1000 nm.

GIXS. Grazing-incidence X-ray scattering (GIXS) were performed at beamline BL25A of the National Synchrotron Radiation Research Center (NSRRC), Taiwan. During the measurements, X-rays were incident on the sample surface at a grazing angle of 0.04°, and the scattering signals were collected in the reflection direction to probe the microstructure of the thin films. For sample preparation, polymer thin films were typically fabricated by spin-coating the polymer solutions onto silicon wafers, followed by thermal annealing at 110 °C for 1 h.

Thermal evaporator. Gold electrodes were thermally evaporated using equipment at the Center for Emerging Material and Advanced Devices, National Taiwan University.

2. Synthesis

1-[(2,2’-Bithiophen)-5-yl]octan-1-one (3)

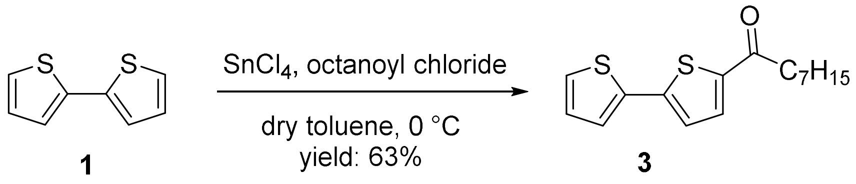

Dry toluene (25 ml) was added into a three-necked round-bottom flask containing 2,2’-bithiophene (4.00 g, 24.06 mmol), and anhydrous $SnCl_4$ (2.67 ml, 22.86 mmol) was added to the solution at 0 °C. After 10 minutes, octanoyl chloride (3.90 ml, 22.86 mmol) was added dropwise via an addition funnel. The mixture was allowed to stir at room temperature for 6 hours. The mixture was quenched by water and extracted with ethyl acetate. The organic layer was collected and evaporated under reduced pressure. Recrystallization from ethanol gave 1-[(2,2’-bithiophen)-5-yl]octan-1-one as a yellow solid (4.20 g, 60%). ^{1}H NMR (600 MHz, $CDCl_3$): δ 7.59 (d, J = 4.0 Hz, 1H), 7.33–7.30 (m, 2H), 7.17 (d, J = 4.0 Hz, 1H), 7.06 (dd, J = 5.0, 3.7 Hz, 1H), 2.88–2.84 (t, J = 7.5 Hz, 2H), 1.78–1.72 (m, 2H), 1.39–1.27 (m, 8H), 0.88 (t, J = 7.0 Hz, 3H). ^{13}C NMR (150 MHz, $CDCl_3$): δ 193.3, 145.3, 142.4, 136.5, 132.5, 128.2, 126.3, 125.5, 124.1, 39.1, 31.7, 29.3, 29.1, 25.0, 22.6, 14.1.

5-Octyl-2,2'-bithiophene (4)

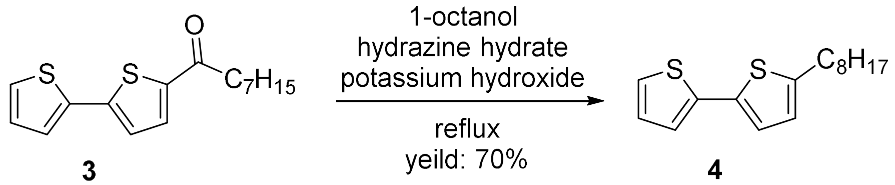

1-[(2,2'-Bithiophen)-5-yl]octan-1-one (16.68 g, 57.03 mmol), 1-octanol (36 ml), hydrazine hydrate (66.72 ml, 1369 mmol) and potassium hydroxide (86.40 g, 1540 mmol) was added into a one-necked round-bottom flask. The mixture was heated to reflux for 6 hours. After being cooled to room temperature, the mixture was extracted with ethyl acetate, dried over anhydrous $MgSO_4$, filtrated and evaporated under reduced pressure. 5-Octyl-2,2'-bithiophene was obtained by column chromatography (silica gel, hexane) as a colorless liquid (11.12 g, 70%). ^{1}H NMR (600 MHz, $CDCl_3$): δ 7.16 (dd, J = 5.1, 1.1 Hz, 1H), 7.09 (dd, J = 3.6, 1.1 Hz, 1H), 6.99–6.97 (m, 2H), 6.67 (m, 1H), 2.78 (t, J = 7.3 Hz, 2H), 1.68 (quintet, J = 7.5 Hz, 2H), 1.42–1.22 (m, 10H), 0.88 (t, J = 7.0 Hz, 3H). ^{13}C NMR (150 MHz, $CDCl_3$): δ 145.4, 138.0, 134.7, 127.6, 124.7, 123.7, 123.4, 123.0, 31.9, 31.6, 30.1, 29.3, 29.2, 29.1, 22.6, 14.1.

5-Octyl-5'-(tri-*n*-butylstannyl)-2,2'-bithiophene (5)

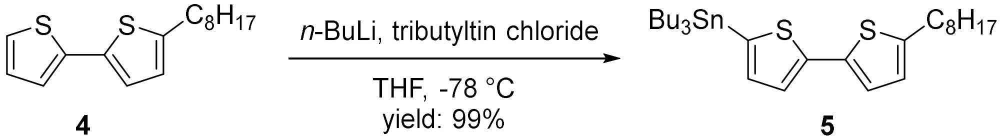

4 5

Dry THF (50 mL) was added into a three-necked round-bottom bottle containing 5-octyl-2,2'-bithiophene (2.78 g, 9.98 mmol) under a nitrogen atmosphere. To the THF solution at −78 °C was added dropwise *n*-butyllithium (11.23 mL, 17.97 mmol, 1.6 M in hexane). After stirring at this temperature for 1 hour, *n*-Bu_3SnCl (4.87 ml, 17.97 mmol) was added. The reaction mixture was allowed to raise to room temperature and stirred overnight. The mixture was quenched by water and extracted with ether. The organic layer was dried over anhydrous $MgSO_4$, filtrated, and evaporated under reduced pressure. Column chromatography (neutral aluminium oxide, hexane) was used to give 5-octyl-5'-(tri-*n*-butylstannyl)-2,2'-bithiophene as a colorless liquid. It was used for the next step without further purification.

1,6-Bis(bithiophene)pyrene (A)

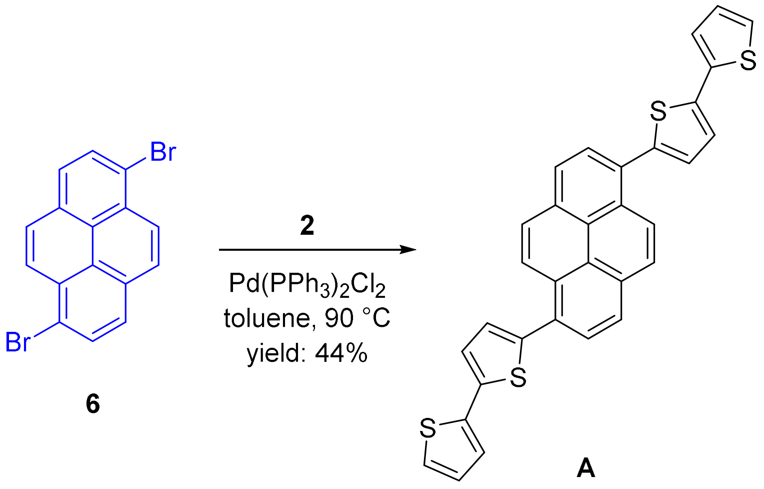

Dry toluene (200 mL) was added into a one-necked round-bottom flask containing 1,6-dibromopyrene (800.00 mg, 2.22 mmol), 5-trimethylstannyl-2,2'-bithiophene (2.181 g, 6.63 mmol), and $Pd(PPh_3)_2Cl_2$ (62.05 mg, 0.0884 mmol). The mixture was stirred at 90 °C for 13 hours. After being cooled to room temperature, the mixture was quenched by water and extracted with dichloromethane. The organic layer was collected and evaporated under reduced pressure. 1,6-Bis(bithiophene)pyrene was obtained by recrystallization from chloroform as a yellow crystal (518.43 mg, 44%). ^{1}H NMR (600 MHz, $CDCl_3$): δ 8.61 (d, J = 9.2 Hz, 2H), 8.20 (d, J = 7.9 Hz, 2H), 8.14 (d, J = 7.9 Hz, 2H), 8.12 (d, J = 9.0 Hz, 2H), 7.34 (d, J = 3.6 Hz, 2H), 7.31 (d, J = 3.6 Hz, 2H), 7.29 (dd, J = 3.5, 1.1 Hz, 2H), 7.28 (dd, J = 5.1, 1.1 Hz, 2H), 7.08 (dd, J = 5.1, 3.5 Hz, 2H). HRMS (MALDI, m/z) calcd for C32H18S4 (M+): 530.0291; found: 530.0300.

1,6-Bis[5-octyl-(2,2'-bithiophen)-5'-yl]pyrene (B)

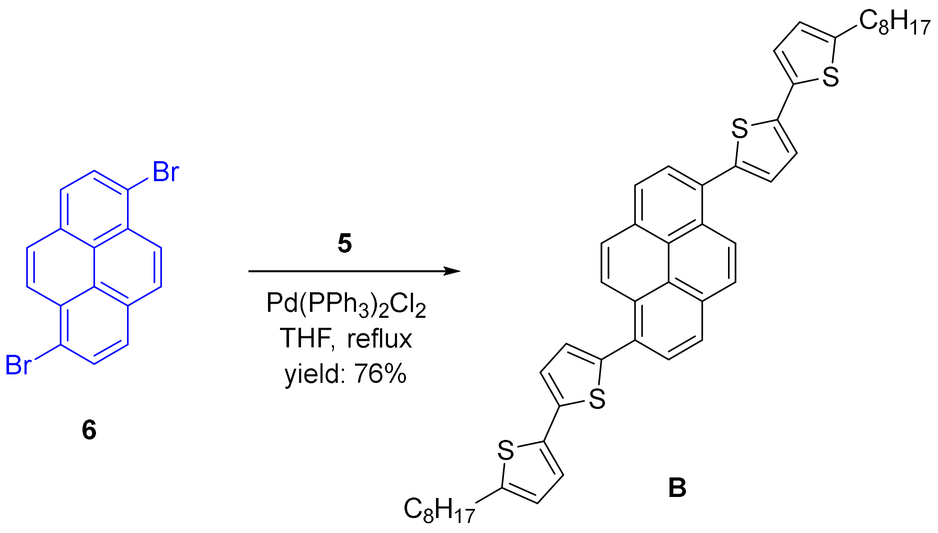

Dry THF (25 mL) was added into a three-necked round-bottom flask containing 1,6-dibromopyrene (500.00 mg, 1.39 mmol), 5-octyl-5’-(tri-*n*-butylstannyl)-2,2’-bithiophene (2.364 g, 4.17 mmol), and $Pd(PPh_3)_2Cl_2$ (100 mg, 0.14 mmol). The mixture was stirred at 70 °C overnight under a nitrogen atmosphere. After being cooled to room temperature, the mixture was quenched by water and extracted with chloroform. The organic layer was dried over $MgSO_4$, filtrated, and evaporated under reduced pressure. 1,6-Bis[5-octyl-(2,2'-bithiophen)-5'-yl]pyrene was obtained by column chromatography (silica gel, chloroform:hexane = 1:9) and recrystallization from chloroform as a dark yellow solid (799.90 mg, 76%). ^{1}H NMR (600 MHz, $CDCl_3$): 8.60 (d, J = 9.2 Hz, 2H), 8.19 (d, J = 7.9 Hz, 2H), 8.12 (d, J = 7.9 Hz, 2H), 8.10 (d, J = 9.4 Hz, 2H), 7.28 (d, J = 3.6 Hz, 2H), 7.25 (d, J = 3.6 Hz, 2H), 7.09 (d, J = 3.6 Hz, 2H), 6.73 (d, J = 3.6 Hz, 2H), 2.83 (t, J = 7.8 Hz, 4H), 1.72 (m, 4H), 1.44–1.28 (m, 20H), 0.89 (t, J = 7.0 Hz, 6H). HRMS (MALDI, m/z) calcd for C48H50S4 (M+): 754.2795; found: 754.2793.

2,7-Bis[5-octyl-(2,2'-bithiophen)-5'-yl]pyrene (C)

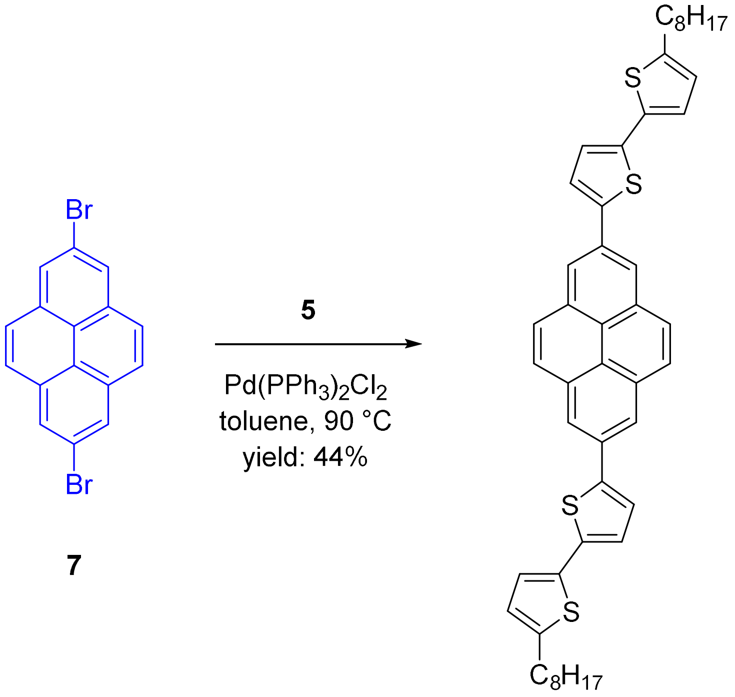

7

C

Dry toluene (25 mL) was added into a three-necked round-bottom flask containing 2,7-dibromopyrene (500.00 mg, 1.39 mmol), 5-octyl-5'-(tri-*n*-butylstannyl)-2,2'-bithiophene (2.364 g, 4.17 mmol), and $Pd(PPh_3)_2Cl_2$ (100 mg, 0.14 mmol). The mixture was stirred at 90 °C for 3 days under a nitrogen atmosphere. After being cooled to room temperature, the mixture was quenched by water and extracted with dichloromethane. The organic layer was dried over $MgSO_4$, filtrated, and evaporated under reduced pressure. 2,7-Bis[5-octyl-(2,2'-bithiophen)-5'-yl]pyrene was obtained by column chromatography (silica gel, chloroform:hexane = 1:9) and recrystallization from 1,2-dichlorobenzene as a yellow solid (600 mg, 57%). ^{1}H NMR (600 MHz, $CDCl_3$): δ 8.36 (s, 4H), 8.08 (s, 4H), 7.51 (d, J = 3.6 Hz, 2H), 7.18 (d, J = 3.8 Hz, 2H), 7.09 (d, J = 3.4 Hz, 2H), 6.73 (d, J = 3.5 Hz, 2H), 2.83 (t, J = 7.8 Hz, 4H), 1.73–1.69 (m, 4H), 1.33 (m, 20H),

0.90 (t, *J* = 7.0 Hz, 6H). HRMS (MALDI, m/z) calcd for C48H50S4 (M+): 754.2795; found: 754.2808.

4,9-Bis[5-octyl-(2,2'-bithiophen)-5'-yl]pyrene (D)

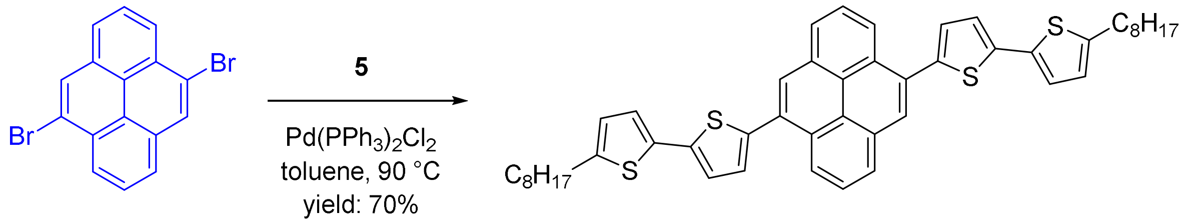

8 D

Dry toluene (25 mL) was added into a three-necked round-bottom flask containing 4,9-dibromopyrene (500.00 mg, 1.39 mmol), 5-octyl-5'-(tri-*n*-butylstannyl)-2,2'-bithiophene (2.364 g, 4.17 mmol), and $Pd(PPh_3)_2Cl_2$ (100 mg, 0.14 mmol). The mixture was stirred at 90 °C for 3 days under a nitrogen atmosphere. After being cooled to room temperature, the mixture was quenched by water and extracted with chloroform. The organic layer was dried over $MgSO_4$, filtrated, and evaporated under reduced pressure. 4,9-Bis[5-octyl-(2,2'-bithiophen)-5'-yl]pyrene was obtained by column chromatography (silica gel, chloroform:hexane = 1:9) and recrystallization from hexane as a brown solid (740 mg, 70%). ^{1}H NMR (600 MHz, $CDCl_3$): δ 8.67 (d, *J* = 8.1Hz, 2H), 8.24 (d, *J* = 7.4 Hz, 2H), 8.23 (s, 2H), 8.04 (t, *J* = 7.7 Hz, 2H), 7.35 (d, *J* = 3.6 Hz, 2H), 7.26 (d, *J* = 3.6 Hz, 2H), 7.10 (d, *J* = 3.5 Hz, 2H), 6.74 (d, *J* = 3.5 Hz, 2H), 2.84 (t, *J* = 7.5 Hz, 4H), 1.73 (m, 4H), 1.44–1.24 (m, 20H), 0.90 (t, *J* = 7.1 Hz, 6H). HRMS (MALDI, m/z) calcd for C48H50S4 (M+): 754.2795; found: 754.2801.

3. TGA

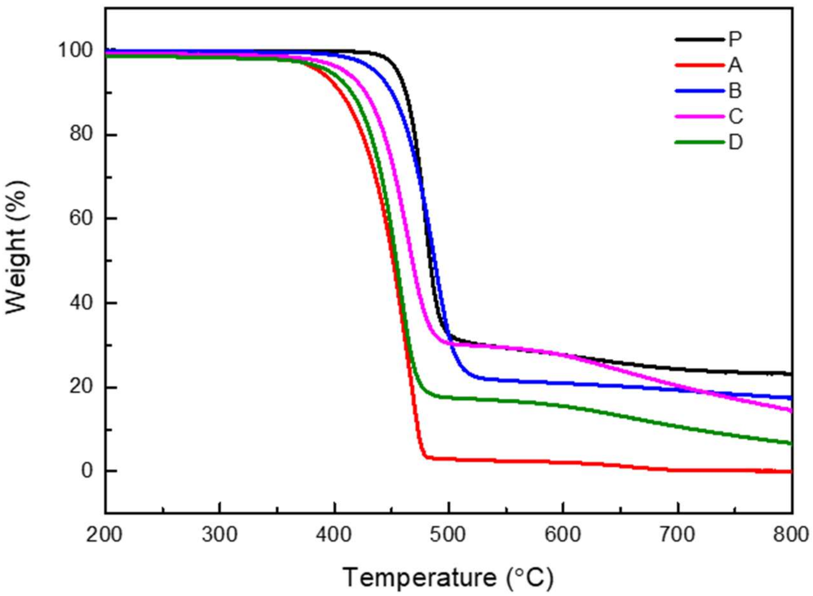

Figure S1. TGA thermograms of **P**, **A**, **B**, **C**, and **D**.

4. UV-vis spectroscopy

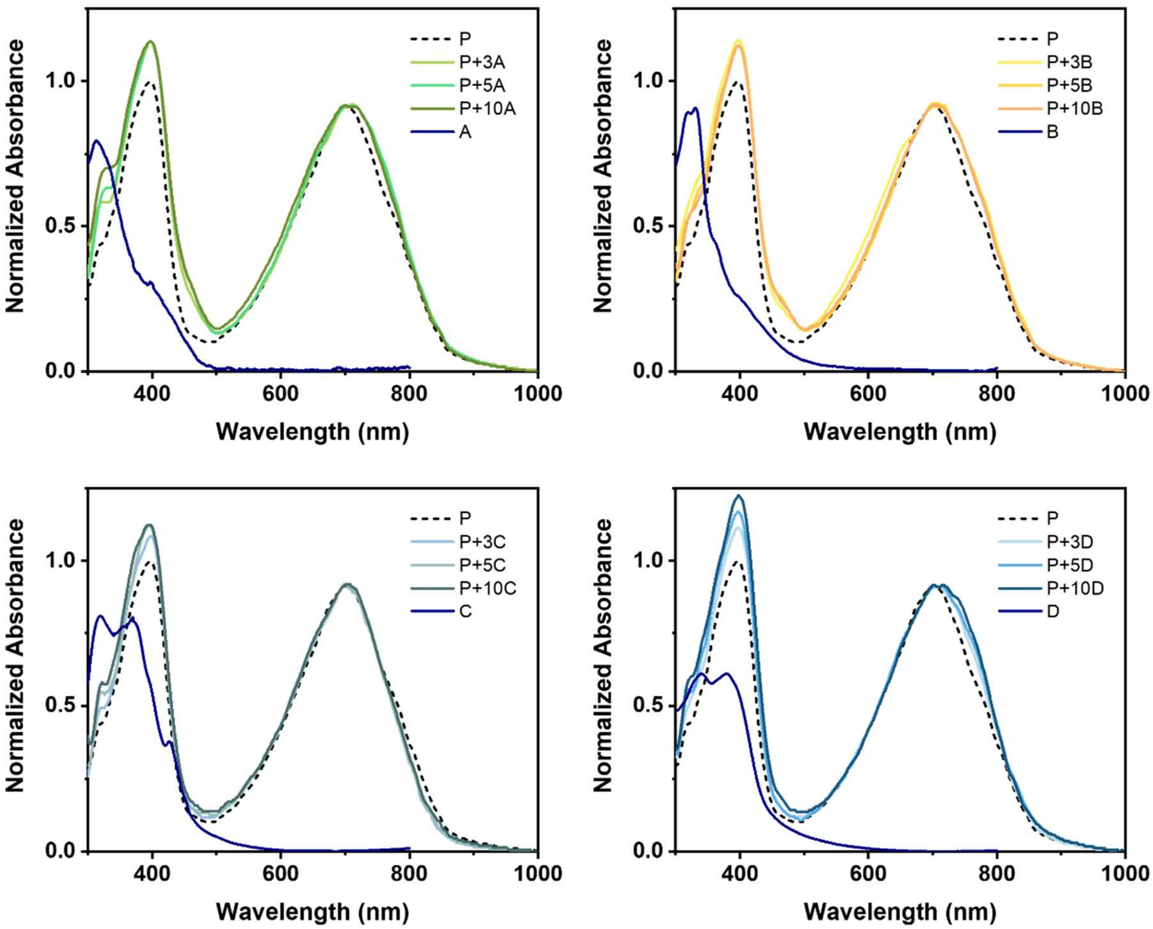

Figure S2. UV-vis absorption spectra in thin films.

5. DSC

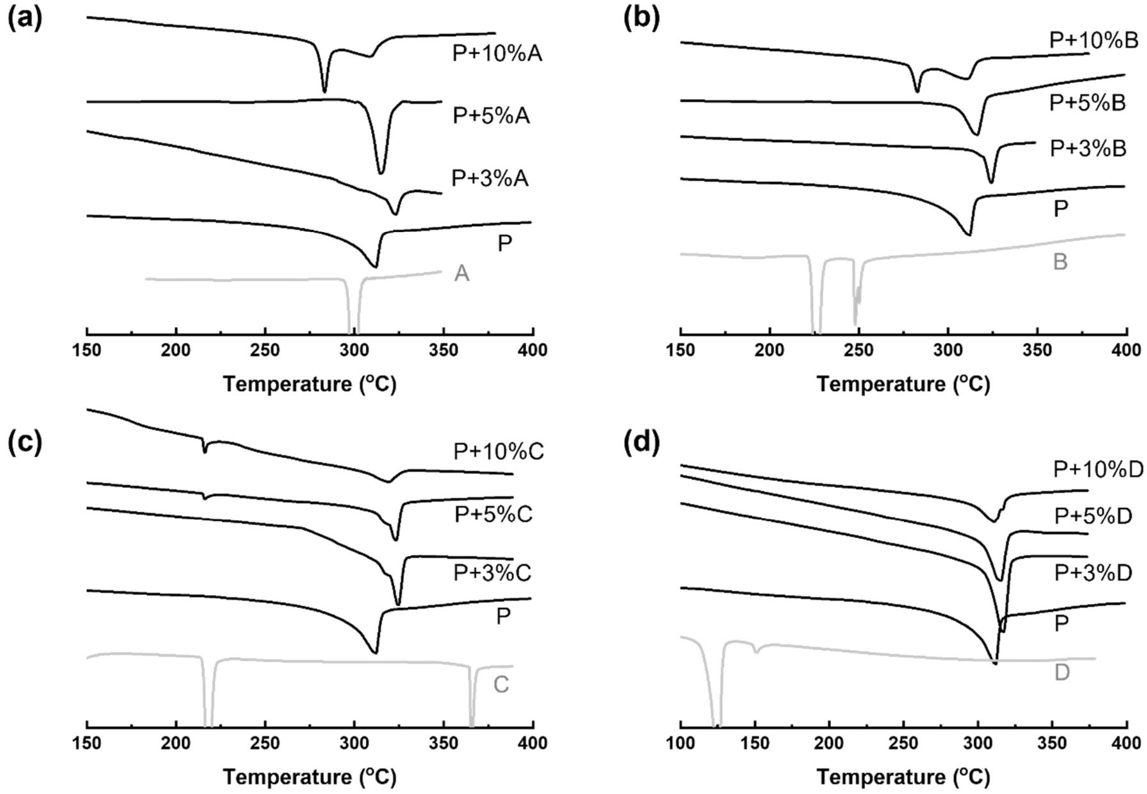

Figure S3. DSC curves for (a) **A** blends, (b) **B** blends, (c) **C** blends, and (d) **D** blends.

Shoulder regions were more visible in the blends of **P**+3%**C**, **P**+5%**C**, **P**+3%**D**, and **P**+5%**D**. Using the previous curve-fitting method, their endothermic DSC curves near the T_m were fitted with a combination of two functions: the first function describes the heat flow of **P**, while the second is a sigmoid curve representing the glass transition of the RAF, yielding the change in heat capacity of the polymer (W_s). After fitting, the W_s values were 0.48 W g^{-1} for **P**+3%**C**, 0.17 W g^{-1} for **P**+5%**C**, 0.36 W g^{-1} for **P**+3%**D**, and 0.40 W g^{-1} for **P**+5%**D**.

$$q_{fit} = W_p \cdot q_p(T - T_{0,p}) - \frac{W_s}{1 + e^{-k_s(T - T_g)}}$$

q_{fit} represents the resulting pattern used to fit a DSC curve, while $q_P(T)$ denotes the heat flow curve of polymer **P**. The parameter set ($T_{0,P}$, W_P, T_g, W_s, k_s) is used for fitting. Here, $T_{0,P}$ and W_P correspond to the shift in melting temperature and the amplification factor for $q_P(T)$, respectively.

T_g is the glass transition temperature, W_s is the change in heat capacity of the polymer, and k_s represents the logistic growth rate or steepness of the curve.

6. ^{1}H NMR spectroscopy

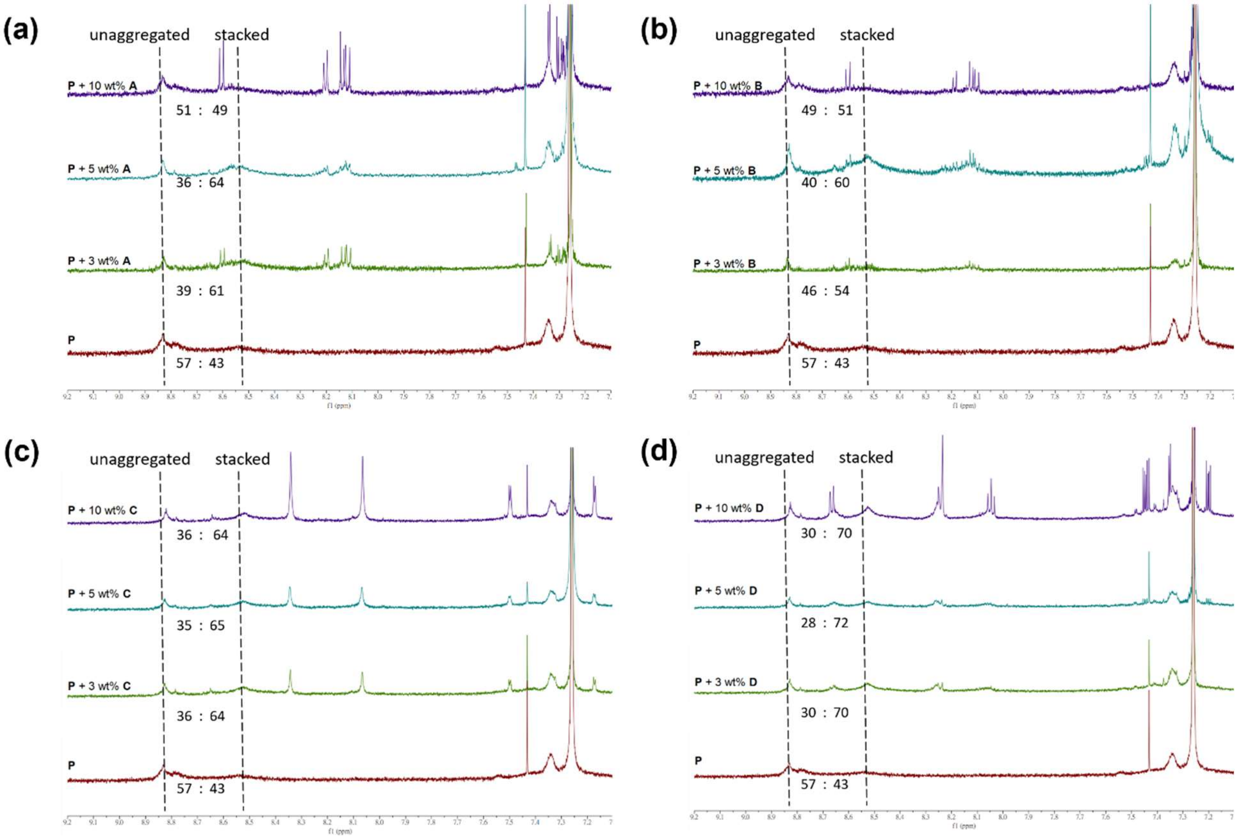

Figure S4. ^{1}H NMR spectra of **P** blended with **A**, **B**, **C**, and **D**.

7. GIXS

(a)

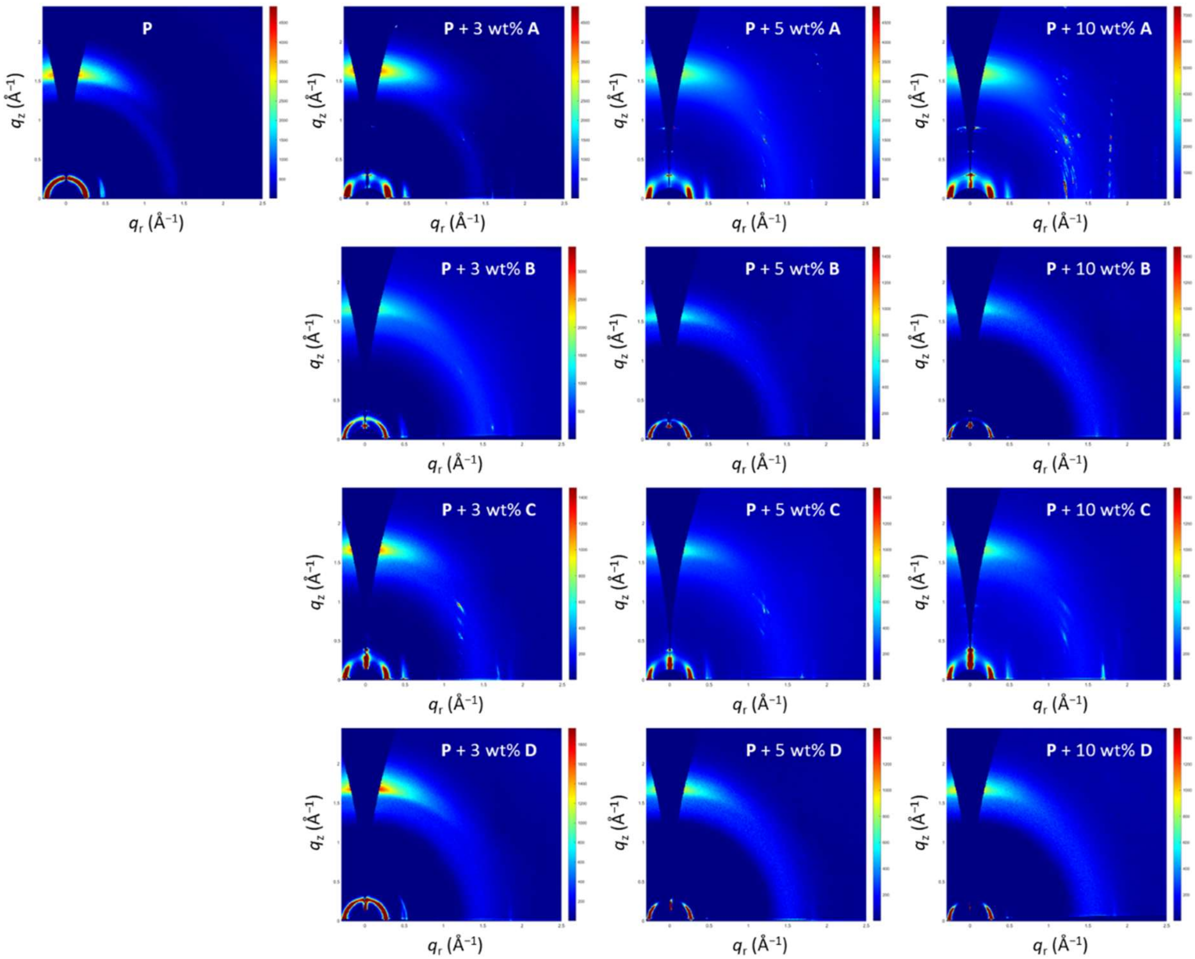

P
P + 3 wt% A
P + 5 wt% A
P + 10 wt% A
P + 3 wt% B
P + 5 wt% B
P + 10 wt% B
P + 3 wt% C
P + 5 wt% C
P + 10 wt% C
P + 3 wt% D
P + 5 wt% D
P + 10 wt% D
q_z (Å^{-1})
q_r (Å^{-1})

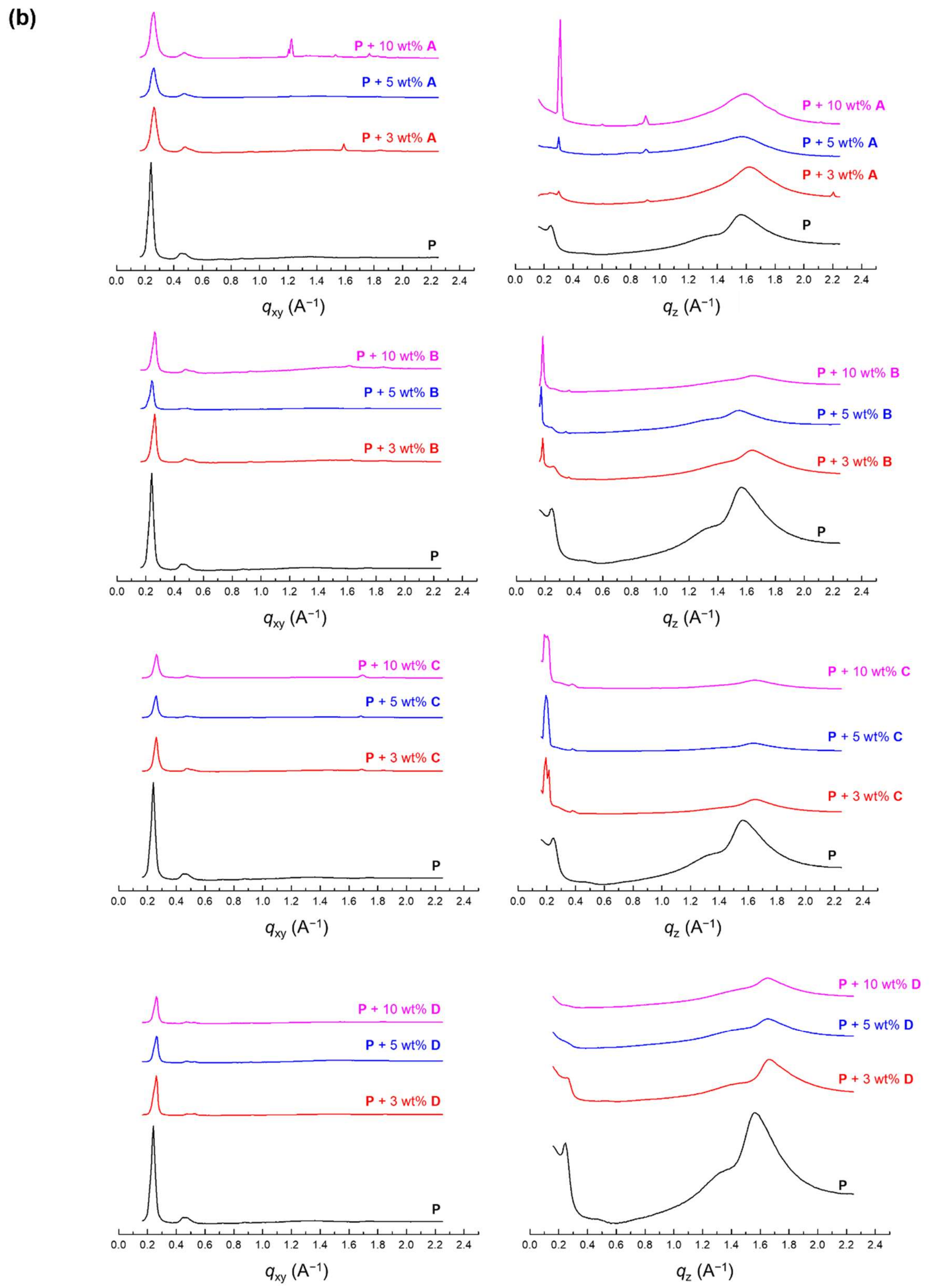

Figure S5. (a) Two-dimensional GIXS images and (b) one-dimensional GIXS patterns.

Table S1. Summary for GIXS results

	Lamellar-Stacking		π-Stacking		Polymer backbone	
	d [Å]	$L_{c,L}$ [Å]	d [Å]	$L_{c,\pi}$ [Å]	d [Å]	$L_{c,B}$ [Å]
**P**	26.3	166	4.0	21	13.5	95
**P**+ 3%**A**	24.1	119	3.9	16	13.2	126
**P**+ 5%**A**	24.4	128	4.0	17	13.3	109
**P**+ 10%**A**	24.5	119	3.9	17	13.3	89
**P**+ 3%**B**	24.3	165	3.8	18	13.2	115
**P**+ 5%**B**	26.0	197	4.0	21	14.1	107
**P**+ 10%**B**	24.2	171	3.8	17	13.2	120
**P**+ 3%**C**	24.2	178	3.8	21	13.2	165
**P**+ 5%**C**	24.4	179	3.8	22	13.3	153
**P**+ 10%**C**	24.0	170	3.8	19	13.2	149
**P**+ 3%**D**	24.4	157	3.7	23	13.2	144
**P**+ 5%**D**	24.1	167	3.8	19	13.3	128
**P**+ 10%**D**	24.2	181	3.8	18	13.3	144

8. DFT calculations

Geometry optimization was carried out using the Gaussian 16 software package at the ωB97XD/6-311G(d,p) level of theory. The ωB97XD functional is a long-range corrected hybrid density functional incorporating empirical dispersion corrections, making it suitable for describing non-covalent interactions. The 6-311G(d,p) basis set was employed for all calculations.

9. OFET

Device fabrication

1) A heavily n-doped silicon wafer with 300 nm silicon dioxide on one side was cut into pieces measuring 1.3 × 1.3 cm^2. The silicon pieces were immersed in piranha solution (H_2SO_4:H_2O_2 = 3:1) for one day. After removal from the solution, they were sequentially washed with deionized water, acetone, and isopropyl alcohol, each with 5 minutes of sonication. The wafers were then gently cleaned with lens tissue and placed in a clean Petri dish.
2) The silicon pieces were treated in a Jelight UV-ozone cleaner for 30 minutes to generate hydroxyl groups on the surface.
3) The treated silicon pieces were immersed in anhydrous toluene (150 mL) containing *n*-octadecyltrichlorosilane (ODTS, 3.75 mL) for one day, followed by washing with hexane, acetone, and isopropyl alcohol. The wafers were then dried.
4) A 1.0 × 1.0 cm^2 polydimethylsiloxane film was placed on the silicon wafer, which was then subjected to UV-ozone treatment in a Jelight cleaner for 3 minutes. After treatment, the wafer was cleaned and dried. The uncovered area of the wafer retained hydroxyl groups, facilitating subsequent thin-film deposition.
5) A polymer solution (chloroform/*o*-dichlorobenzene, 98:2 w/w) at a concentration of 8 mg mL^{-1} was spin-coated onto the ODTS-modified silicon wafers at 1000 rpm for 60 seconds to form thin films. The films were thermally annealed at 110 °C for 1 hour.
6) Gold source and drain electrodes (40 nm thick) were deposited onto the thin films using a thermal evaporator at a rate of 0.25 Å s^{-1} through a shadow mask. The resulting devices had a channel width of 1 mm and a channel length of 0.1 mm.

7) Device isolation was achieved using a diamond knife to ensure independent operation of each device.

8) Electrical characterization of the devices was performed under a nitrogen atmosphere using an Agilent Technologies B1500A semiconductor parameter analyzer.

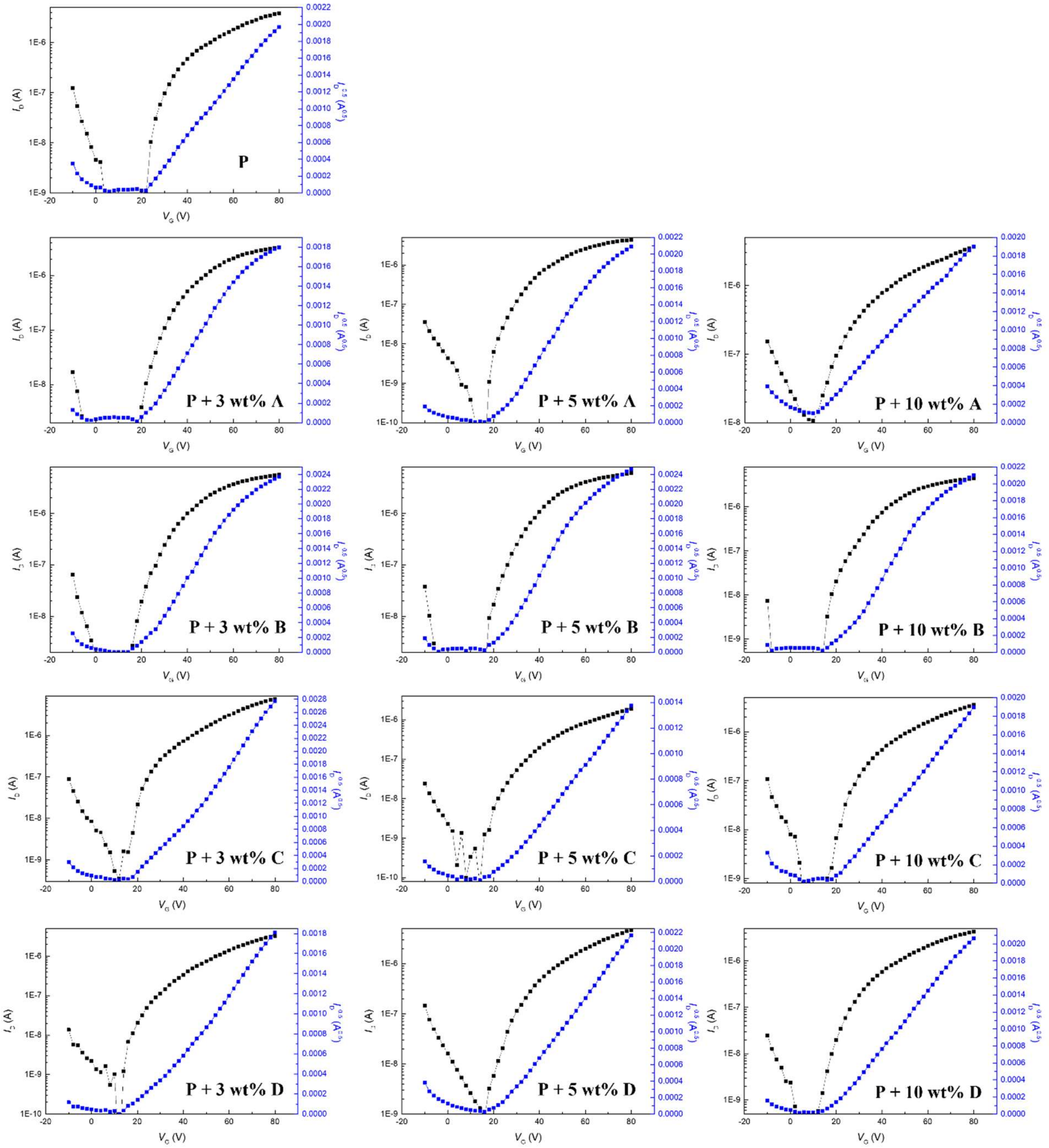

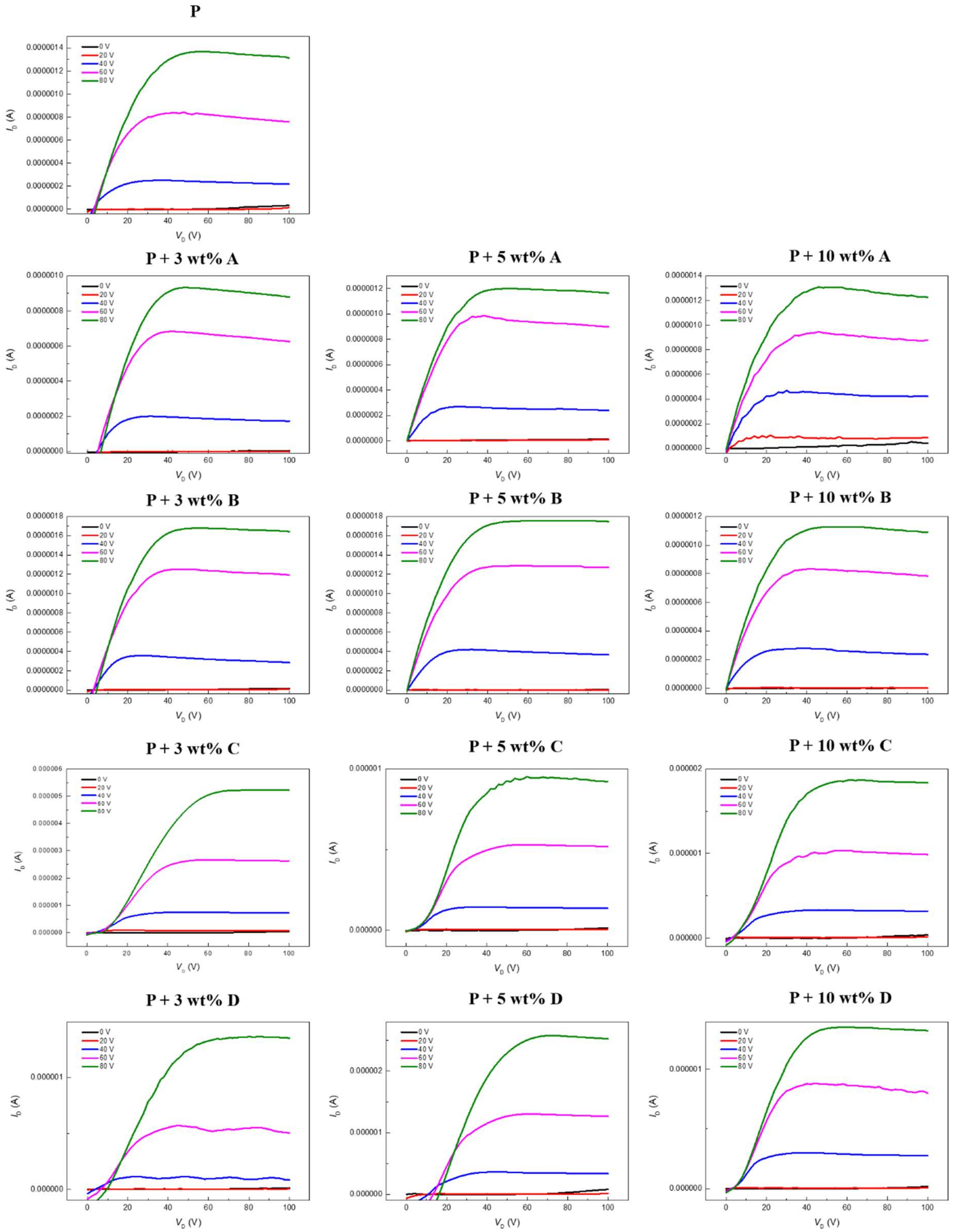

Figure S6. Representative transfer and output curves of OFETs.

Table S2. Summary for OFET results

**Blend**	**μ_e^{max} ($cm^2V^{-1}S^{-1}$)**	**V_{th} (V)**	**μ_e^{avg} ($cm^2V^{-1}S^{-1}$)**	**V_{th}^{avg} (V)**	**On/off ratio**
**P**	0.027	17.8	0.020 ± 0.006	20.05	3.0×10^4
**P**+ 3**A**	0.037	21.9	0.030 ± 0.005	21.46	2.7×10^5
**P**+ 5**A**	0.049	22.1	0.038 ± 0.006	20.94	1.7×10^4
**P**+ 10**A**	0.020	11.3	0.013 ± 0.004	6.710	3.6×10^2
**P**+ 3**B**	0.076	19.1	0.048 ± 0.014	16.04	3.0×10^5
**P**+ 5**B**	0.061	20.8	0.053 ± 0.007	18.66	1.0×10^5
**P**+ 10**B**	0.039	21.7	0.025 ± 0.007	15.36	1.9×10^4
**P**+ 3**C**	0.054	25.6	0.043 ± 0.009	24.25	2.3×10^5
**P**+ 5**C**	0.028	22.0	0.020 ± 0.007	19.53	2.5×10^4
**P**+ 10**C**	0.021	17.9	0.019 ± 0.002	14.90	6.3×10^3
**P**+ 3**D**	0.023	22.7	0.018 ± 0.005	25.80	6.7×10^4
**P**+ 5**D**	0.029	18.4	0.022 ± 0.005	20.33	3.0×10^3
**P**+ 10**D**	0.025	14.5	0.017 ± 0.006	17.29	1.7×10^4

10. Linear regression analysis

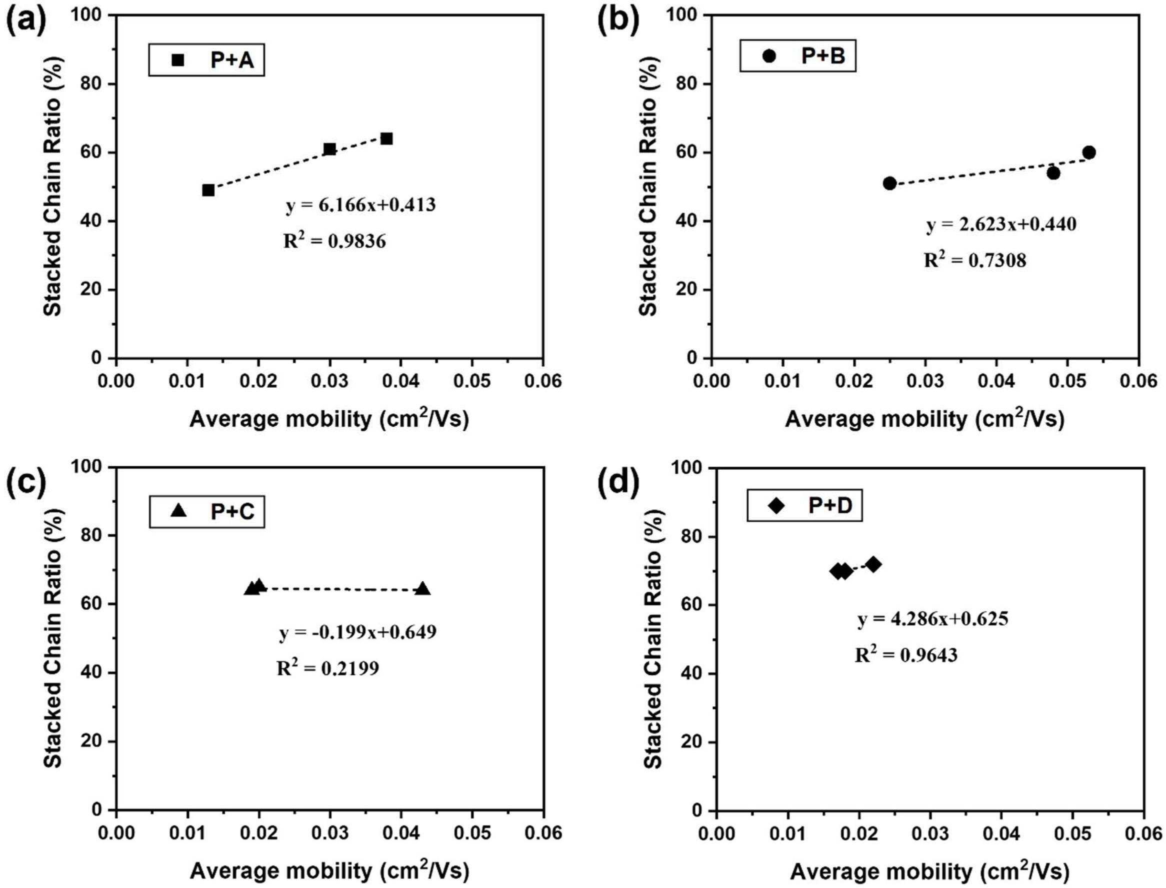

Figure S7. Linear regression analyses of the average μ_e versus the stacked-chain proportion for (a) **A** blends, (b) **B** blends, (c) **C** blends, and (d) **D** blends.

11. NMR and mass spectra

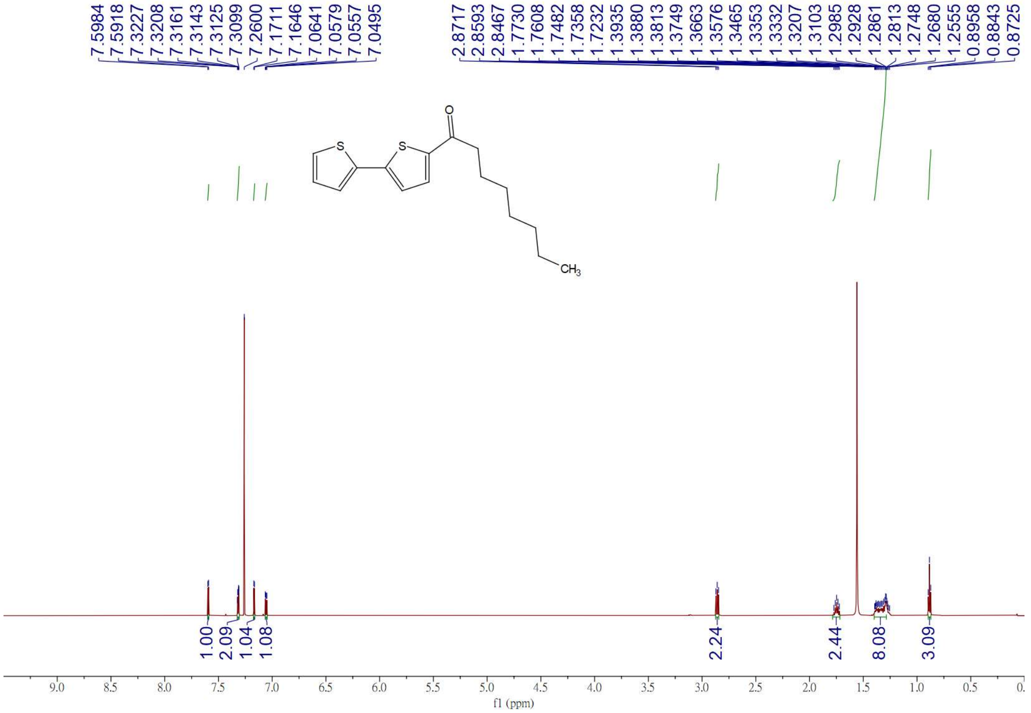

Figure S8. ^{1}H NMR spectrum of 1-[(2,2'-bithiophen)-5-yl]octan-1-one (**3**).

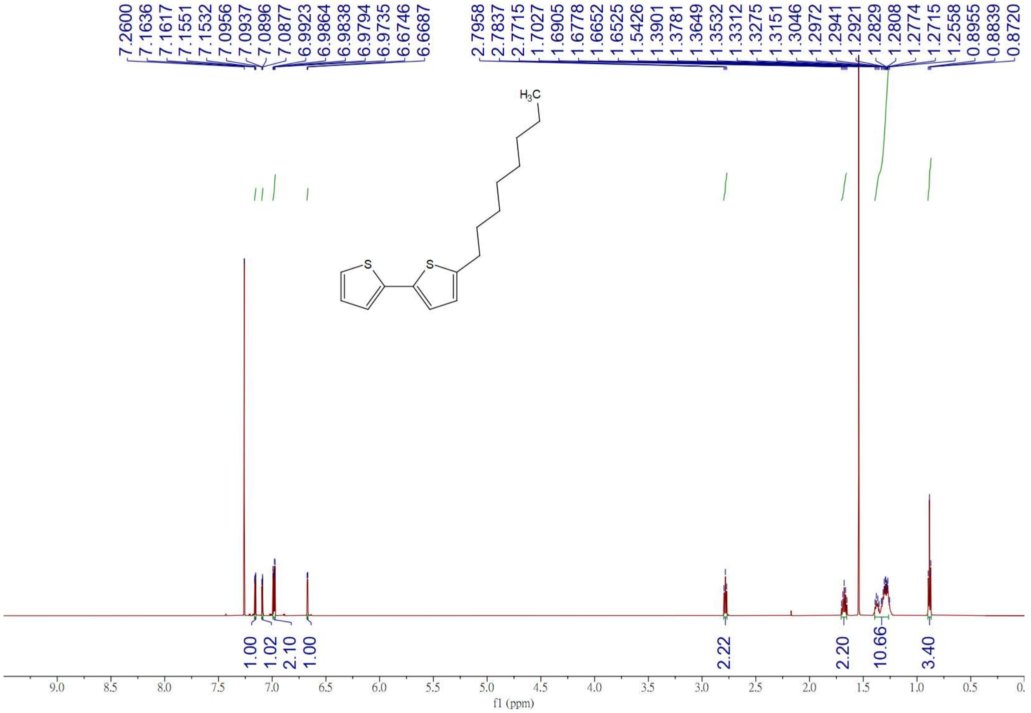

Figure S9. ^{1}H NMR spectrum of 5-octyl-2,2'-bithiophene (**4**).

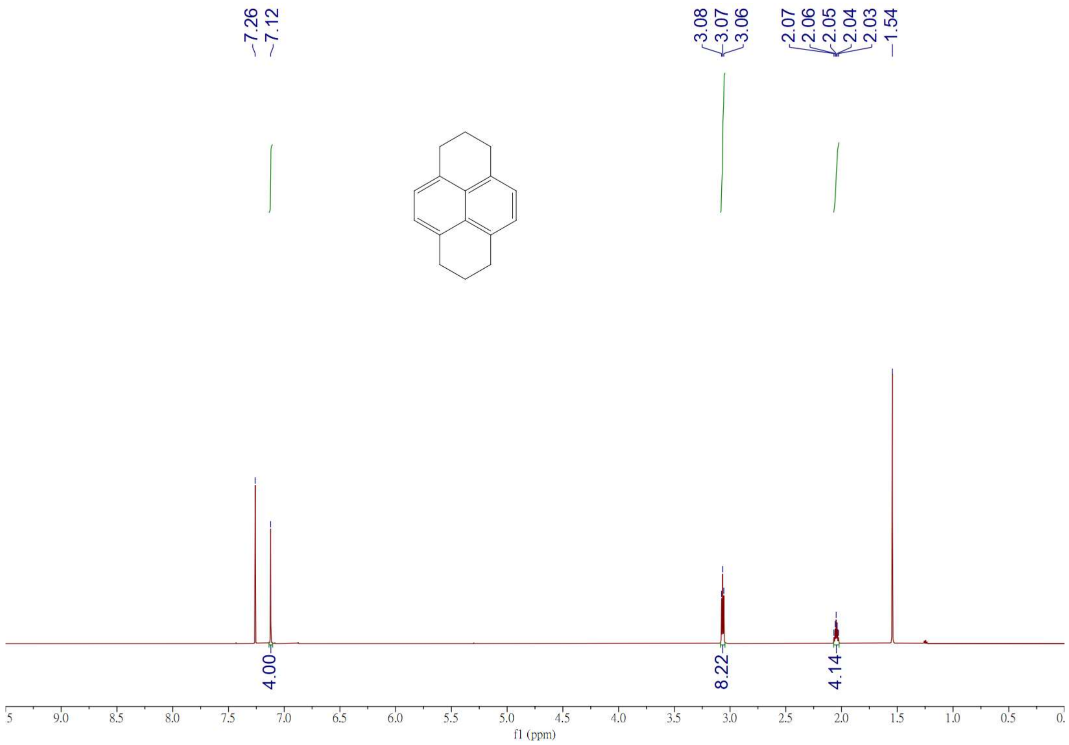

Figure S10.^{1}H NMR spectrum of 1,2,3,6,7,8-hexahydropyrene.

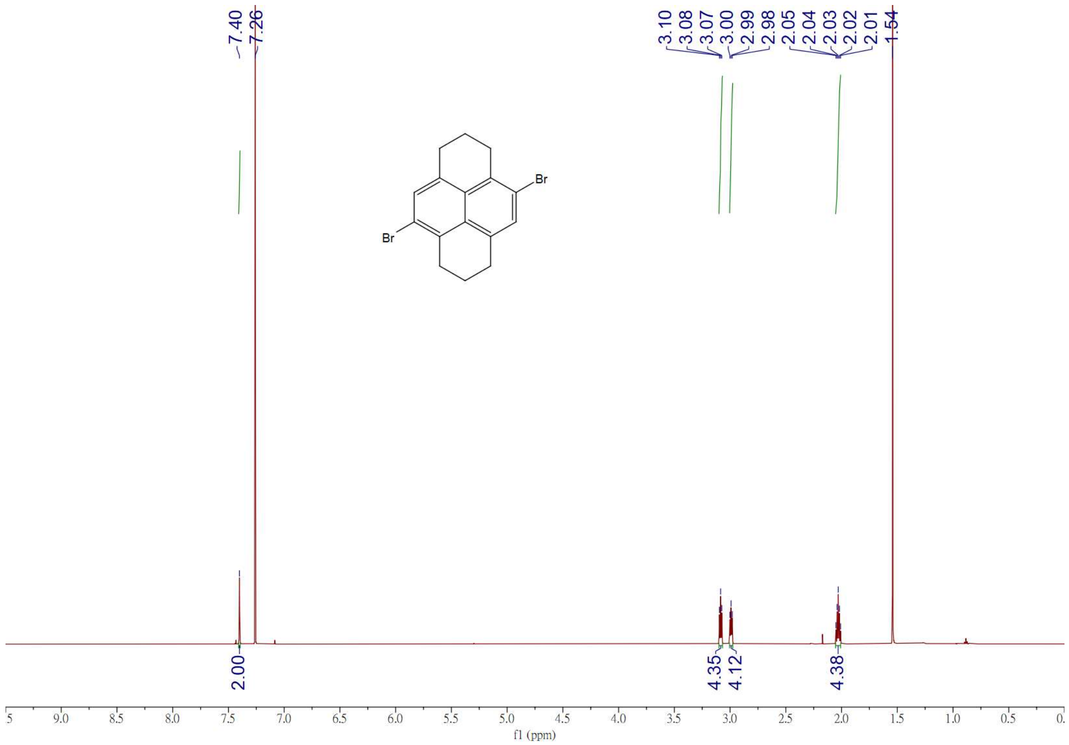

Figure S11. ^{1}H NMR spectrum of 4,9-dibromo-1,2,3,6,7,8-hexahydropyrene.

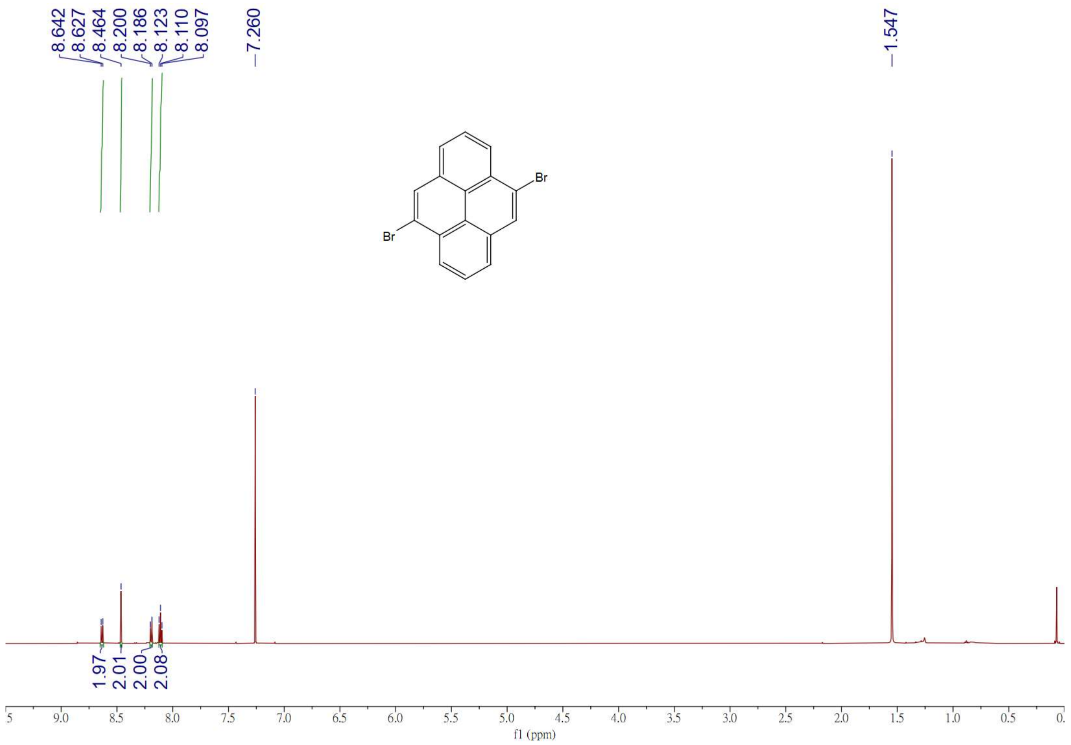

Figure S12. ¹H NMR spectrum of 4,9-dibromopyrene (**8**).

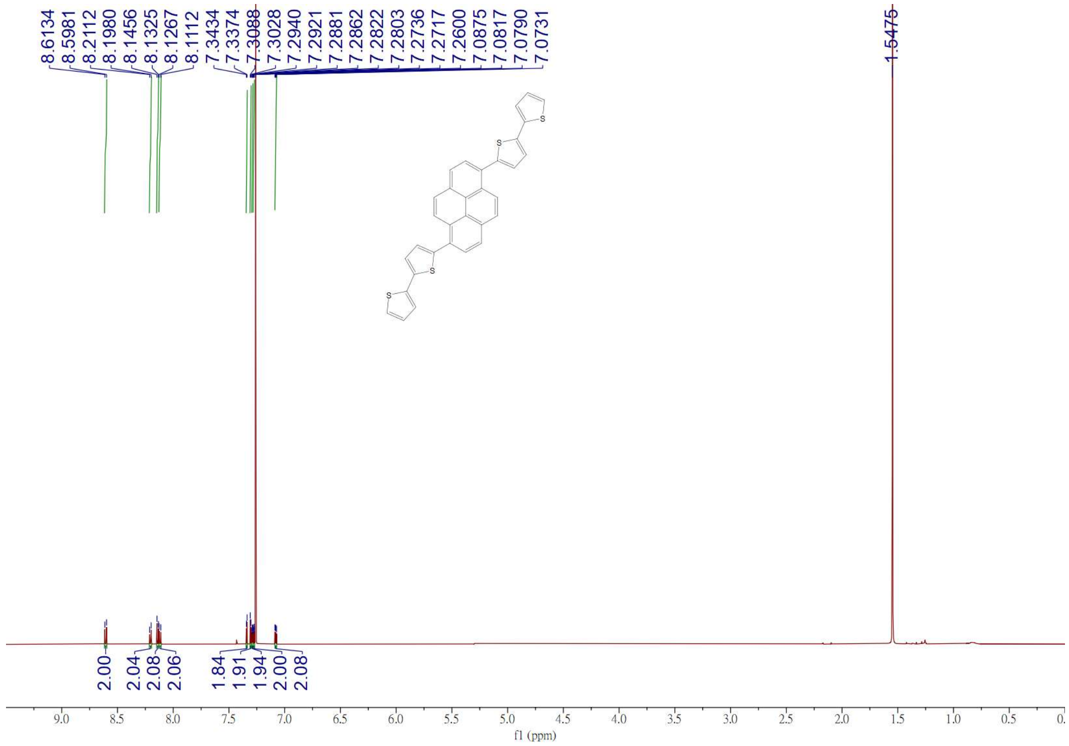

Figure S13. ^{1}H NMR spectrum of 1,6-bis(bithiophene)pyrene (**A**).

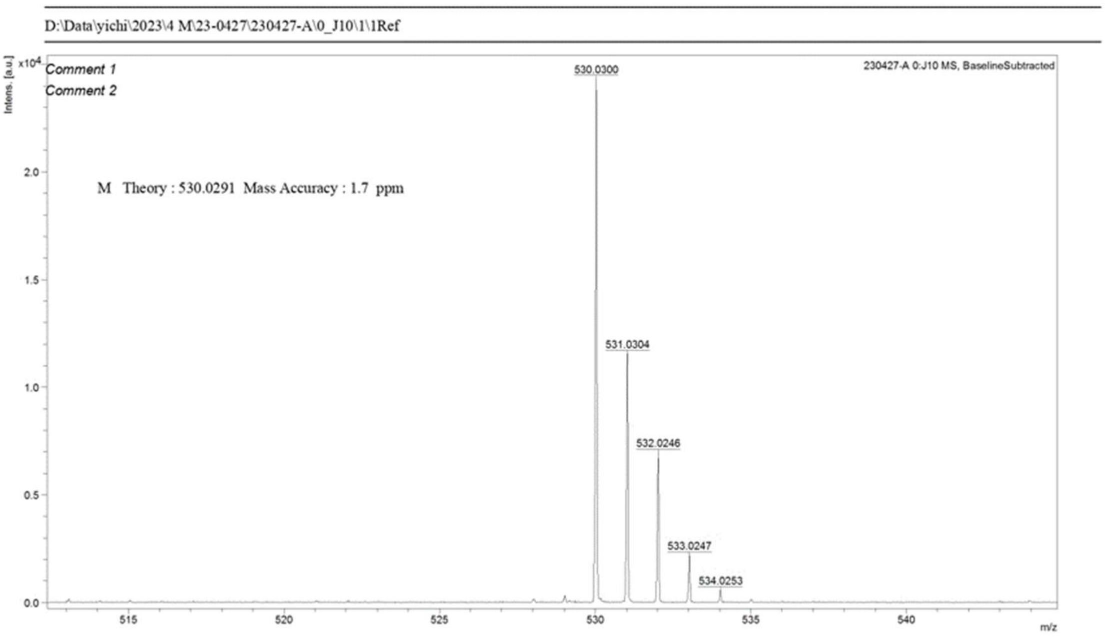

Figure S14. HR MALDI-TOF mass spectrum of 1,6-bis(bithiophene)pyrene (**A**).

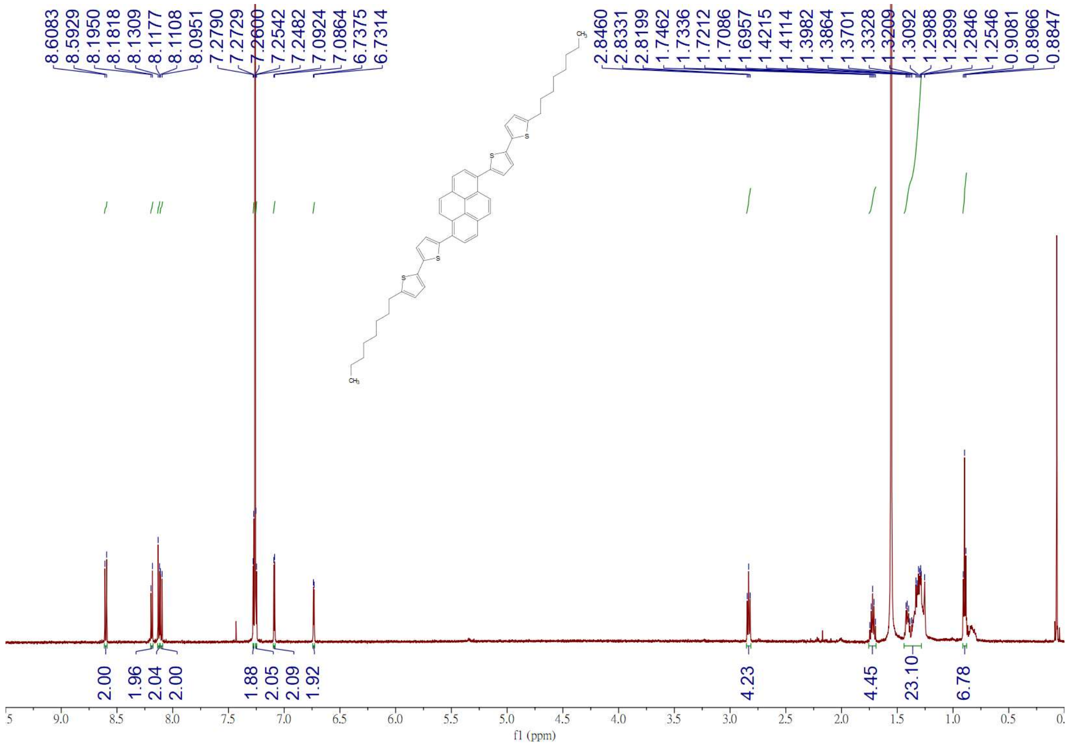

Figure S15. ^{1}H NMR spectrum of 1,6-bis[5-octyl-(2,2'-bithiophen)-5'-yl]pyrene (**B**).

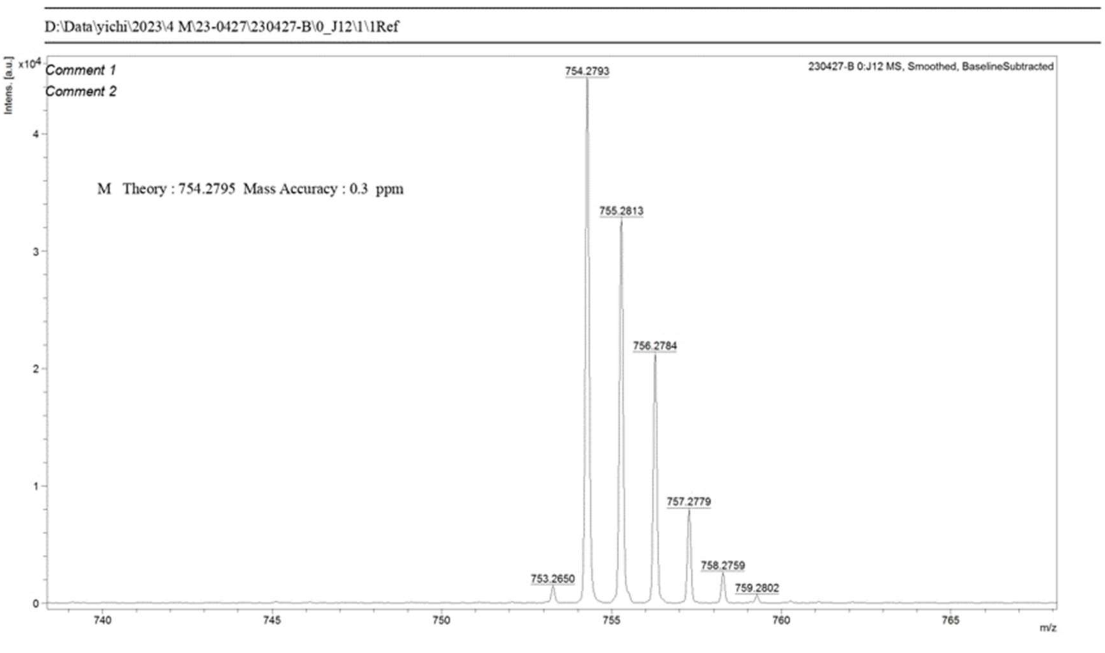

Figure S16. HR MALDI-TOF mass spectrum of 1,6-bis[5-octyl-(2,2'-bithiophen)-5'-yl]pyrene (**B**).

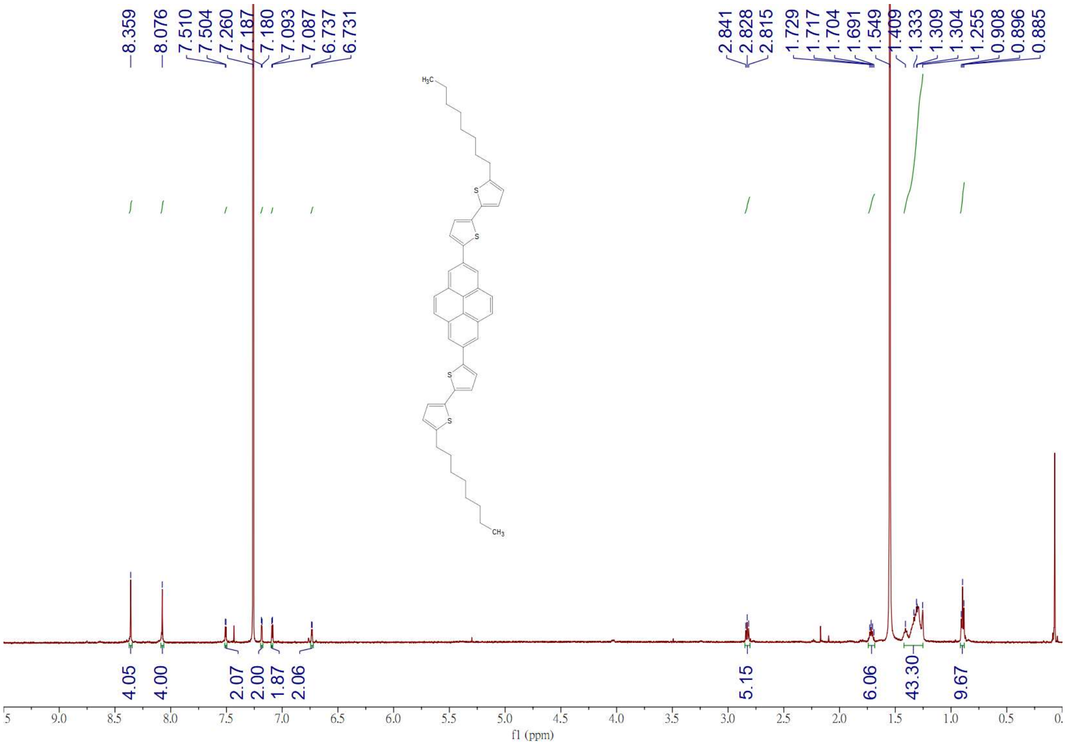

Figure S17. ^{1}H NMR spectrum of 2,7-bis[5-octyl-(2,2'-bithiophen)-5'-yl]pyrene (**C**).

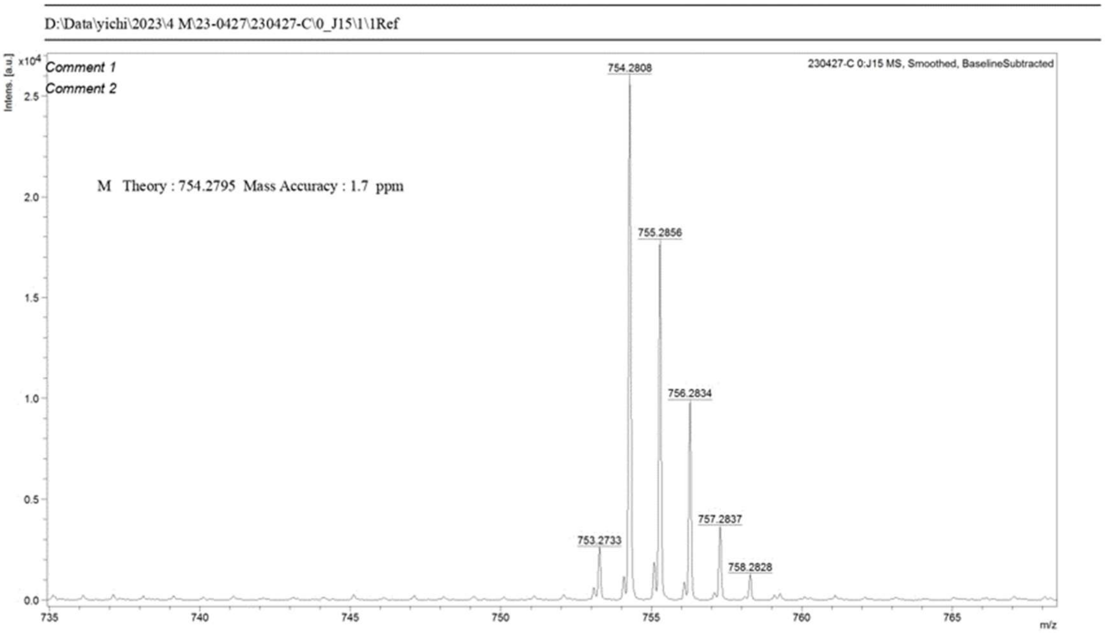

Figure S18. HR MALDI-TOF mass spectrum of 2,7-bis[5-octyl-(2,2'-bithiophen)-5'-yl]pyrene (**C**).

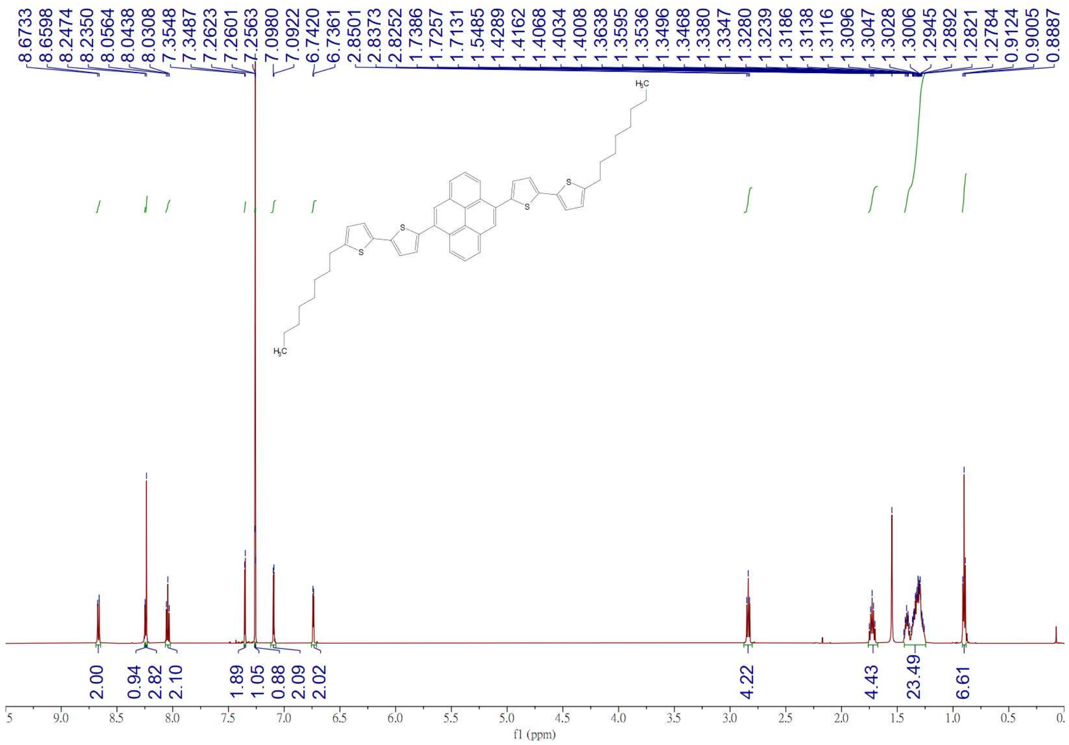

Figure S19. ^{1}H NMR spectrum of 4,9-bis[5-octyl-(2,2'-bithiophen)-5'-yl]pyrene (**D**).

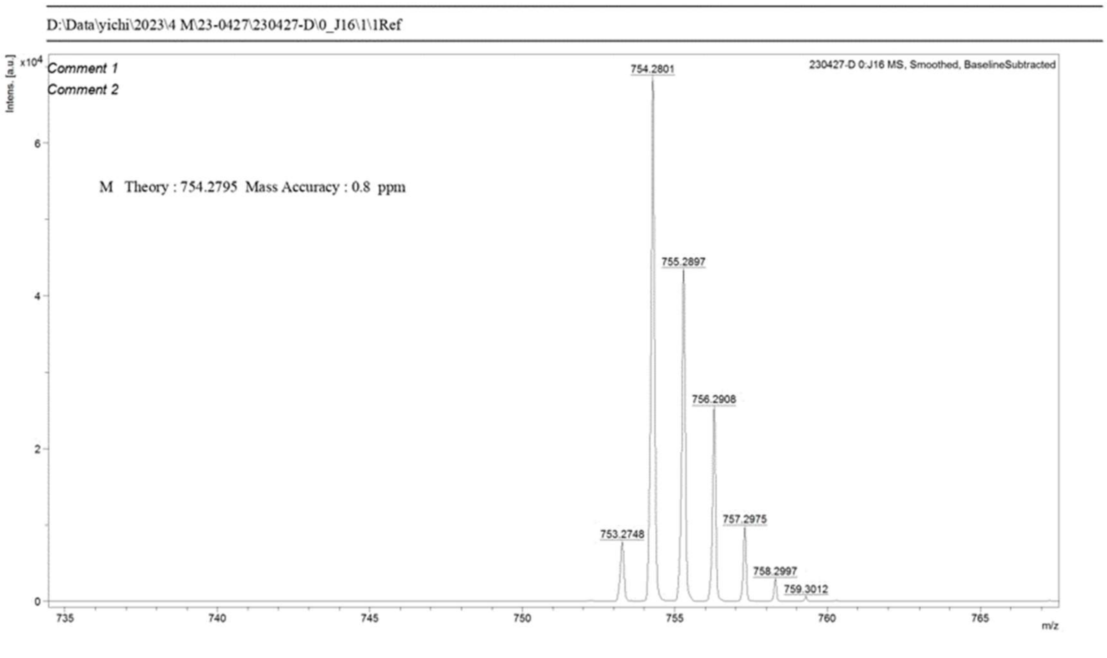

Figure S20. HR MALDI-TOF mass spectrum of 4,9-bis[5-octyl-(2,2'-bithiophen)-5'-yl]pyrene (**D**).
